# Synergistic mechanotherapy and sonopermeation guided by mathematical modeling for solid tumor treatment

**DOI:** 10.3389/fddev.2025.1549098

**Published:** 2025-06-24

**Authors:** Marina Koutsi, Triantafyllos Stylianopoulos, Fotios Mpekris

**Affiliations:** ^1^ Cancer Biophysics Laboratory, Department of Mechanical and Manufacturing Engineering, University of Cyprus, Nicosia, Cyprus; ^2^ Cancer Genetics, Therapeutics & Ultrastructural Pathology Department, The Cyprus Institute of Neurology and Genetics, Nicosia, Cyprus

**Keywords:** computational modeling, nano-immunotherapy, breast cancer, tumor microenvironment, drug delivery

## Abstract

The progression of tumors and their response to treatment are significantly influenced by the presence of elevated mechanical solid stress. This solid stress compresses intratumoral blood vessels, leading to reduced blood flow (hypoperfusion) and insufficient oxygen levels (hypoxia), both of which hinder the delivery of oxygen and therapeutic agents. As a result, these conditions promote tumor growth, resistance to treatment, and ultimately undermine the effectiveness of therapies. To address these challenges, strategies like mechanotherapeutics and ultrasound sonopermeation have been developed to enhance blood flow and improve drug delivery to tumors. Mechanotherapy aims to reduce the mechanical stress and stiffness within tumors, helping to decompress vessels and restore normal perfusion. Ultrasound sonopermeation temporarily increases the permeability of blood vessel walls in a non-invasive manner, boosting blood flow and improving the delivery of therapeutic drugs. Here, we developed a mathematical model to explore the combined effects of mechanotherapeutics and sonopermeation on optimizing nano-immunotherapy efficacy. The model integrates complex interactions between key components involved in tumor progression, including tumor cells, immune cells, and vascular elements such as endothelial cells, angiopoietins, and vascular endothelial growth factor. To assess the model’s validity, its predictions for key parameters, including tumor volume, functional vascular density, and hypoxia levels, were compared with experimental data, demonstrating a strong correlation, and confirming the accuracy of the mathematical framework. Furthermore, we carried out a parametric analysis to establish critical guidelines aimed at optimizing both the sequence and timing of experimental procedures. Specifically, we investigated the therapeutic outcomes of two treatment scenarios: applying sonopermeation first, followed by nano-immunotherapy, and *vice versa*. Also, we determined the optimal time interval between the application of sonopermeation and the commencement of the combined nano-immunotherapy regimen to maximize therapeutic efficacy.

## 1 Introduction

The tumor microenvironment (TME) in solid tumors is a complex system consisting not only of malignant cells but also stromal cells and extracellular matrix (ECM) components, which together influence tumor growth and progression (Erkan et al., 2012; Jain 2008a; Jain 2013; Stylianopoulos, Munn, and Jain, 2018; Vakoc et al., 2009). In certain types of cancers, particularly those with high desmoplastic activity, such as breast cancer and various sarcoma subtypes, the TME often becomes fibrotic as tumors grow. This fibrosis is marked by an increased production of ECM components, notably collagen and hyaluronan, leading to the formation of a tumor mass with increased stiffness and altered mechanical properties (Mpekris et al., 2024). As the tumor expands, the dense aggregation of cancer cells, stromal elements, and ECM, along with its invasive growth into surrounding tissues, generates mechanical forces known as solid stress. These forces act both within the tumor and at the tumor-host tissue interface (Jain, Martin, and Stylianopoulos, 2014; Stylianopoulos, 2017). Solid stress plays a pivotal role in tumor progression and impacts on the effectiveness of therapeutic interventions (Kalli et al., 2023). High levels of solid stress can compress intratumoral blood vessels, causing them to collapse and lose their functionality. This vascular impairment leads to reduced oxygen and nutrient delivery, resulting in hypoperfusion and hypoxia (Padera et al., 2004; Stylianopoulos et al., 2012; Stylianopoulos et al., 2013). The impaired blood flow poses a major challenge to the efficient delivery of systemically administered therapies into the tumor, while the accompanying hypoxic conditions can accelerate tumor progression and promote treatment resistance through multiple mechanisms (Jain, 2014).

A therapeutic approach to improve blood flow and enhance perfusion within tumor tissues involves the use of mechanotherapeutics. These agents work to normalize the physical properties of tumors by alleviating stiffness and reducing internal mechanical stresses (Sheridan, 2019). By targeting specific components of the extracellular matrix, such as collagen and hyaluronan, or focusing on Cancer-Associated Fibroblasts (CAFs), mechanotherapeutics can relieve pressure on compressed blood vessels, improving both perfusion and the intratumoral distribution of therapeutic agents (Mpekris, Papageorgis, et al., 2017; Panagi et al., 2020; Papageorgis et al., 2017; Polydorou et al., 2017; Mpekris et al., 2021; Chauhan et al., 2013; Voutouri et al., 2021; Panagi et al., 2022; Mpekris et al., 2023; Kalli and Stylianopoulos, 2024). Experimental and mathematical evidence has highlighted the effectiveness of tranilast, an anti-fibrotic drug, as a mechanotherapeutic agent (Mpekris et al., 2018). Tranilast operates by reducing stress within the tumor, which decompresses blood vessels, enhances vascular density, and improves the delivery of treatment agents (Papageorgis et al., 2017). Additionally, ketotifen, an antihistamine drug, has shown dual functionality within the TME, acting as both a mechanomodulator and an immunomodulator, particularly in sarcomas and breast cancers (Mpekris et al., 2024; Charalambous et al., 2024; Neophytou et al., 2025; Panagi et al., 2024). Despite their benefits, mechanotherapeutics have limitations. They are capable of alleviating compression in only a subset of blood vessels within the tumor, falling short of achieving complete decompression across the entire vascular network (Chauhan et al., 2013). This partial efficacy is primarily due to the heterogeneous nature of the tumor microenvironment. For instance, losartan reduces solid stress by targeting cancer-associated fibroblasts and lowering the expression of signaling molecules like TGF-β1 and CCN2, which are responsible for producing collagen and hyaluronan. These two matrix components synergistically create the solid stress that compresses blood vessels (Chauhan et al., 2013; Stylianopoulos et al., 2012; Voutouri and Stylianopoulos, 2018). Losartan is most effective in tumors rich in both collagen and hyaluronan. In contrast, in less desmoplastic tumors, the effect of losartan and other mechanotherapeutics is limited (Diop-Frimpong et al., 2011; Chauhan et al., 2013; Voutouri et al., 2023; Kalli et al., 2024; Mpekris et al., 2023). Furthermore, mechanotherapeutics are often repurposed drugs that even though approved for clinical use, they are associated with their own adverse effects. For example, losartan is a potent anti-hypertensive drug. Therefore, there are limitations in increasing the dose of these drugs to decompress more vessels.

Another promising method to improve drug delivery in solid tumors is the so called sonopermeation, which combines ultrasound with microbubbles. By temporarily increasing the permeability of blood vessel walls, sonopermeation facilitates the enhanced penetration of therapeutic agents into tumor tissues. This approach has shown potential in overcoming biological barriers and improving treatment efficacy (Soulheim et al., 2021; Snipstad et al., 2021). Moreover, sonopermeation has been observed to reduce intratumoral solid stress, thereby improving blood flow and drug distribution; however, the underlying mechanisms remain poorly understood (Snipstad et al., 2018; Snipstad et al., 2021). Studies have demonstrated that ultrasound, when applied in conjunction with microbubbles, achieves greater therapeutic effectiveness compared to conventional nano- and chemo-therapeutic strategies (Carpentier et al., 2016; Dimcevski et al., 2016; Kotopoulis et al., 2013; Lin et al., 2012; Olsman et al., 2020; Snipstad, Morch, et al., 2021). Notably, recent research has highlighted the potential of combining sonopermeation with mechanotherapy, revealing synergistic effects that further enhance therapeutic outcomes (Mpekris et al., 2024). Despite its promise, mathematical modeling of sonopermeation for drug delivery in solid tumors remains in its infancy. Existing models have primarily focused on the role of microbubbles within blood vessels (Hosseinkhah et al., 2013; Cowley and McGinty, 2019), often overlooking perfusion and drug delivery challenges posed by the TME and lacking experimental validation. Recent advances, however, have produced a more comprehensive mathematical model exploring the ability of low-intensity ultrasound to inhibit cancer stem cell growth and spread (Blanco et al., 2025).

Building on this foundation, we developed a mathematical framework to investigate the combined effects of mechanotherapy and sonopermeation on tumor treatment. This model integrates insights from previous computational studies (Mpekris et al., 2015; Mpekris, Baish, et al., 2017; Mpekris et al., 2020; Mpekris et al., 2018; Zhao et al., 2019) and has been designed to optimize the combined application of mechanotherapeutics and sonopermeation. Specifically, it incorporates key factors, such as the effect of ketotifen, a mechanotherapeutic agent, on the TME and the dynamics of sonopermeation. Model predictions were validated against published experimental data (Neophytou et al., 2025), confirming the accuracy and reliability of the framework. Additionally, a parametric analysis was conducted to assess the significance of the order in which sonopermeation and nano-immunotherapy are administered, as well as determining the optimal time interval that should elapse between the application of sonopermeation and the initiation of nano-immunotherapy treatment, thereby elucidating the most effective timing for achieving optimal therapeutic outcomes.

## 2 Materials and methods

### 2.1 Overview of the mathematical model

**FIGURE 1:**
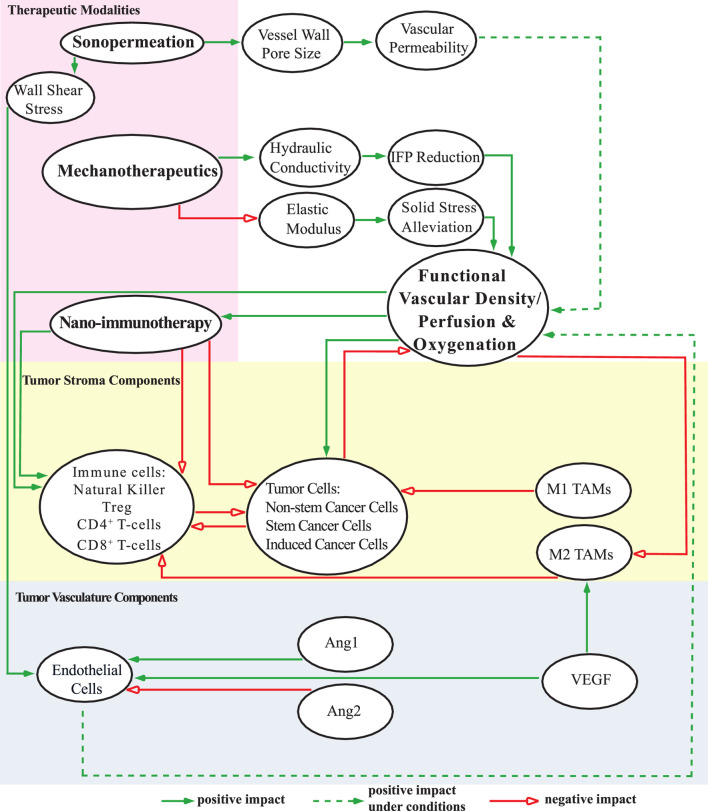
A schematic diagram depicts the components of the mathematical model and their interconnections. The model includes distinct cell populations (represented in the yellow region), tumor angiogenic factors (depicted in the blue region), and various therapeutic strategies, each influencing the TME (highlighted in the pink region) in unique ways. This comprehensive visualization illustrates the interactions among individual components of the model, as well as the potential combinations of these components, shedding light on their collective impact on functional vascular density, perfusion, and oxygenation levels in the system under study. These effects are categorized as positive, negative, or conditionally positive depending on the circumstances.

Figure 1 provides a schematic representation of the tumor’s various components incorporated into the mathematical model, along with their interrelations. The equations, assumptions, and foundational principles of the model are detailed in the Supplementary Material (SM) Appendix. The model captures the complex interactions between key components involved in tumor progression (Figure 1), including:

i) Tumor cell populations: non-stem cancer cells (CCs), stem cancer cells (SCCs), and treatment-induced cancer cells (ICCs) (Mpekris et al., 2020);
ii) Immune cells: NK cells, CD8^+^ T-cells, CD4^+^ T-cells, regulatory T-cells (Tregs), and tumor-associated macrophages (TAMs);
iii) Tumor vasculature components: endothelial cells, angiopoietins (Ang), and vascular endothelial growth factor (VEGF).

**FIGURE 2:**
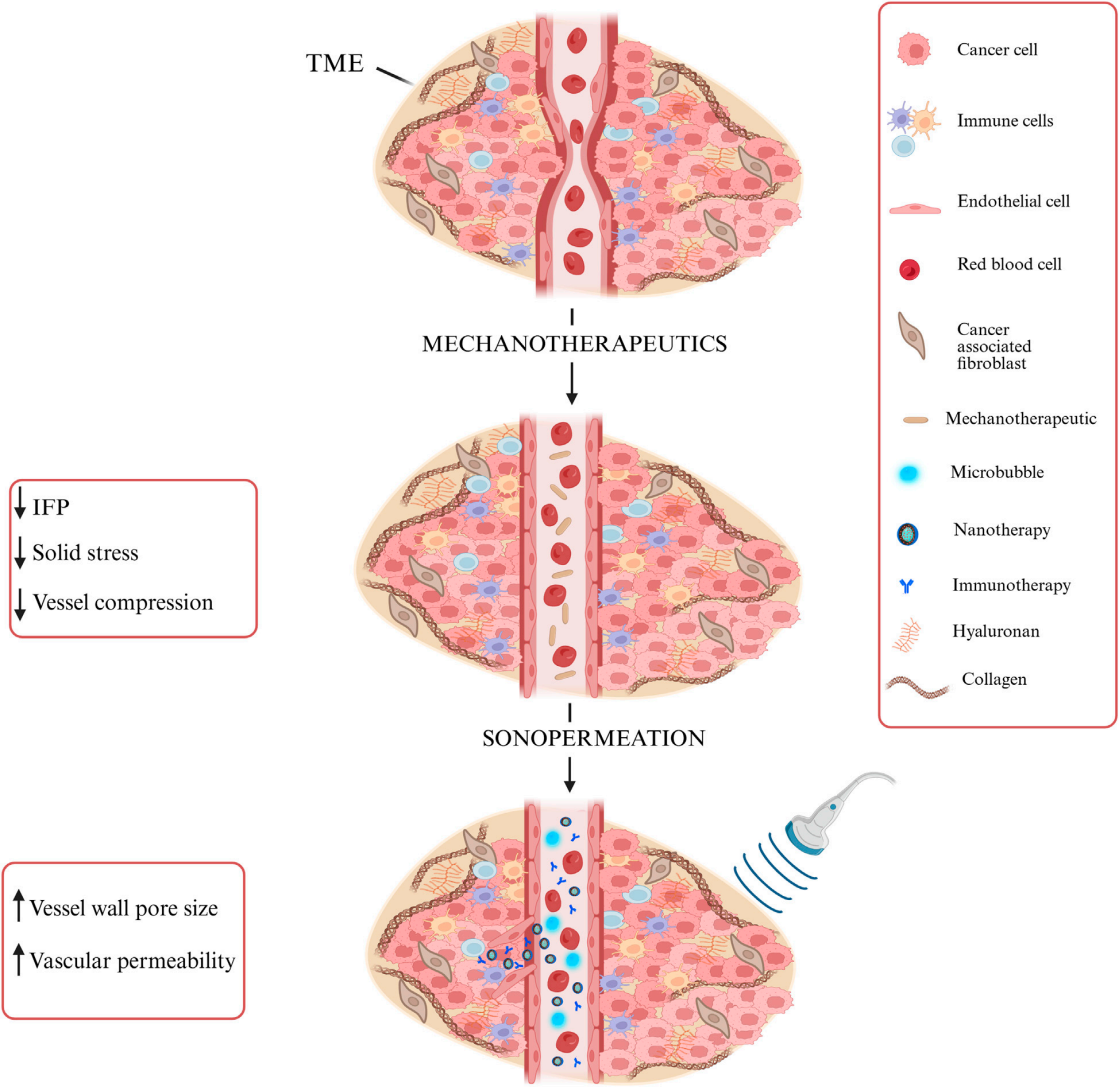
Schematic representation of vessel decompression and perfusion enhancement via Mechanotherapeutics and Sonopermeation. Mechanotherapeutics enhance perfusion by reducing interstitial fluid pressure and solid stress and decompressing vessels. Sonopermeation combined with microbubbles increases vascular permeability and enhances functional vascular density, thereby improving drug delivery. Created with BioRender.com .

The model also accounts for tumor perfusion, oxygenation, and drug delivery, particularly nano-immunotherapy. Sonopermeation enhances vascular permeability by increasing vessel wall pore size, potentially improving functional vascular density (Snipstad et al., 2018; Mpekris et al., 2024). Microbubbles inserted during sonopermeation induce wall shear stress, which influences endothelial cell proliferation and apoptosis (Dolan et al., 2011). Mechanotherapeutics are critical for modulating both the fluid and solid phases of the TME. In the fluid phase, mechanotherapeutics significantly enhance tumor hydraulic conductivity, thereby reducing interstitial fluid pressure (IFP). This reduction improves tumor perfusion (Mpekris et al., 2024; Panagi et al., 2024). In the solid phase, mechanotherapeutics reduce the elastic modulus, alleviating solid stress. This stress relief decompresses blood vessels, leading to increased functional vascular density and improved perfusion (Charalambous et al., 2024; Mpekris et al., 2024; Panagi et al., 2024). The mechanism described above is illustrated in Figure 2, which schematically represents vessel decompression and perfusion enhancement mediated by mechanotherapeutics and sonopermeation.

Enhancement in functional vascular density significantly improves the efficacy of nano-immunotherapy, thereby facilitating the suppression of non-stem cancer cells, stem-like cancer cells, and induced cancer cells (Mpekris et al., 2024). The increased therapeutic concentration achieved with nanomedicine promotes immunogenic cell death (Jain, 2005; Jain, 2008) and enhances the overall effectiveness of immunotherapy (Mpekris et al., 2015; Panagi et al., 2020). This improvement is associated with an elevated CD4^+^/CD8^+^ T-cell ratio (Huang et al., 2013). The synergistic effects of nano-immunotherapy further stimulate the recruitment of effector CD8^+^ T-cells while simultaneously reducing the prevalence of regulatory T-cells (Tregs), thereby fostering a robust anti-tumor immune response (Lu et al., 2017). Additionally, increased tumor oxygenation supports both tumor and immune cell populations and induces a phenotypic shift in tumor-associated macrophages (TAMs) from an immunosuppressive M2 phenotype to an immunostimulatory M1 phenotype (Mpekris, Baish, et al., 2017; de Pillis, Radunskaya, and Wiseman, 2005; Mahlbacher et al., 2018). Tumor cell proliferation, however, leads to substantial compression of the surrounding vasculature, reducing perfusion and impairing immune cell activity within the tumor microenvironment (Griffon-Etienne et al., 1999; Padera et al., 2004). Enhanced proliferation of immune cells amplifies tumor cell eradication, with M1-like TAMs exerting strong tumoricidal effects, while M2-like TAMs inhibit immune effector cell activity, thereby promoting immune suppression and creating an immunosuppressive environment (Goel et al., 2011; Goel, Fukumura, and Jain, 2012).

In terms of tumor vascular components, angiogenesis—a critical process for the formation of new blood vessels—is driven by the proliferation of endothelial cells, thereby improving overall perfusion (Mpekris et al., 2020). Elevated vascular endothelial growth factor (VEGF) levels are associated with an increased prevalence of M2-like TAMs and a heightened rate of endothelial cell proliferation (Carmeliet and Jain, 2011). High concentrations of angiopoietin-2 (Ang2), however, destabilize existing vessels by inhibiting endothelial cell production. This destabilization is counteracted by angiopoietin-1 (Ang1), which stabilizes the vasculature and supports endothelial cell production (Holash et al., 1999; Lobov, Brooks, and Lang, 2002).

In support of these mechanisms described above, SM Appendix, Supplementary Figure S2 presents the computational framework developed to evaluate therapeutic outcomes achieved through mechanotherapy and sonopermeation in solid tumors. This schematic complements Figure 1 by detailing the model’s underlying assumptions, key input parameters, and the governing equations used in the mathematical formulation—each referenced by equation number. It also outlines the numerical methods employed to solve these equations and highlights the key output variables used to assess treatment efficacy.

### 2.2 Drug transport mechanisms

#### 2.2.1 Nanomedicine transport equations

We hypothesized that the delivery of nanomedicine is described by three distinct states (Equations 1–3): (i) the nanoparticle carrier encapsulating the chemotherapeutic agent (c_n_), (ii) the chemotherapeutic agent freely diffusing within the tumor interstitial space (c_f_), and (iii) the chemotherapeutic substance internalized by the cells (c_int_) (Olive et al., 2009). Consequently, the transport of the drug within the interstitial space can be mathematically represented as follows (Stylianopoulos and Jain, 2013):

$$\frac{\partial c_{n}}{\partial t}+\nabla \cdot \left(c_{n}v^{f}\right)=D_{n}\nabla^{2}c_{n}+Q_{sta}-k_{el}c_{n}$$ (1)

$$\frac{\partial c_{f}}{\partial t}+\nabla \cdot \left(c_{f}v^{f}\right)=D_{f}\nabla^{2}c_{f}+{\alpha k}_{el}c_{n}-k_{\mathrm{int}}c_{f}$$ (2)

$$\frac{\partial c_{\mathrm{int}}}{\partial t}+\nabla \cdot \left(c_{\mathrm{int}}v^{s}\right)=k_{\mathrm{int}}c_{f}-k_{\deg}c_{\mathrm{int}}$$ (3)

In this context D_n_ and D_f_ represent the diffusion coefficients of the nanoparticle carrier and the chemotherapeutic agent, respectively, as they diffuse through the tumor interstitial space. The rate constants k_el_, k_int_ and k_deg_ correspond to the release of the chemotherapy from the nanoparticle, the internalization of the drug by cells, and the degradation rate of the chemotherapeutic agent, respectively. Additionally, α indicates the number of chemotherapy molecules encapsulated within a single nanocarrier. The terms v^f^ and v^s^ denote the velocities of the fluid and solid phase within the TME, respectively. Detailed information about v^f^ and v^s^ can be found in the SM Appendix under  Supplementary Equations (S18–S26). For the purposes of this study, the nanomedicine utilized is Doxil. The term Q_sta_, appearing on the right side of Equation 1, describes the transport of the nanocarrier across the tumor vessel wall. This transport process is mathematically defined using Starling’s approximation, (Stylianopoulos and Jain, 2013):

$$Q_{\text{sta}}=P_{\text{er}}S_{V}\left(C_{\text{iv}}-c_{n}\right)+L_{p}S_{V}\left(P_{V}-p_{i}\right)\left(1-\sigma_{f}\right)C_{\text{iv}}$$ (4)

In this formulation, L_p_ represents the hydraulic conductivity of the vessel wall, C_iv_ = exp (-(t-t_0_)/k_d_) describes the vascular concentration of the administered drug, which follows a bolus injection profile. Here, t_0_ refers to the time of drug administration, and k_d_ corresponds to the decay constant associated with blood circulation. The parameter σ_f_ denotes the reflection coefficient. The vascular conductivity L_p_ is defined as a function of the pore radius of the vessel wall, while the parameters P_er_ and σ_f_ are determined based on the ratio of the drug’s molecular radius to the pore radius of the vessel wall (Deen, 1987). A comprehensive explanation of the methodology and calculations used to derive these parameters is provided in the SM Appendix.

#### 2.2.2 Immune checkpoint inhibitors equations

Immunotherapy is incorporated into our mathematical model via the inclusion of immune checkpoint antibodies, which, in specific therapeutic approaches, can be employed simultaneously to augment treatment effectiveness (Mpekris et al., 2020). Within this framework, the influence of anti-PD-1 immune checkpoint inhibition is denoted as an increase in the source term for CD8^+^ T-cells i.e., the term σ_Τ8_, while the use of anti-CTLA-4 is characterized by an elevation in the mortality rate of regulatory T-cells, $m_{\text{reg}}$ (Mpekris et al., 2020). Moreover, the anti-PD-1 antibody is also incorporated into our computational framework as a free pharmacological agent $c_{f_{i}}$ , and this integration is demonstrated through the mathematical formulation presented in Equation 5.

$$\frac{\partial c_{f_{i}}}{\partial t}+\nabla \cdot \left(c_{f_{i}}v^{f}\right)=D_{f_{i}}\nabla^{2}c_{f_{i}}+Q_{{\text{sta}}_{i}}-k_{\deg_{i}}c_{\text{fi}}$$ (5)

In this context, $D_{f_{i}}$ represents the diffusion coefficient of the immune checkpoint antibody within the tumor interstitial space, while $k_{\deg_{i}}$ denotes the rate constant associated with the degradation of the anti-PD-1 antibody. The term $Q_{{\text{sta}}_{i}}$ , appearing on the right-hand side of Equation 5, accounts for the transport of the immune checkpoint antibody across the tumor vessel wall, as defined by Starling’s approximation. It is important to note that the principles described in Equation 4 are similarly applicable to the transport dynamics of the anti-PD-1 antibody, which is capable of diffusing through the interstitial space.

#### 2.2.3 Modeling the impact of mechanotherapeutic ketotifen

Within this mathematical framework, the mechanotherapeutic agent ketotifen has been incorporated to alleviate stress in solid tumors, leading to a notable enhancement in the overall efficacy of nano-immunotherapy (Mpekris et al., 2024; Neophytou et al., 2025). The administration of ketotifen has significant implications, including a reduction in tumor stiffness and a notable enhancement of vascular perfusion, thereby improving the overall functionality of blood vessels. Experimental evidence demonstrates that, within 3 days of ketotifen treatment, tumor stiffness decreases by approximately 50% (Mpekris et al., 2024; Charalambous et al., 2024; Panagi et al., 2024). In addition, ketotifen effectively lowers interstitial fluid pressure, facilitates improved tumor perfusion, and substantially enhances the efficacy of drug delivery (Mpekris et al., 2024). In our computational model, the effects of ketotifen are simulated by reducing the tumor’s shear modulus and increasing its hydraulic conductivity. These changes result in a significant decrease in interstitial fluid pressure and an increase in functional vascular density.

Specifically, in the model we assume that ketotifen induces a linear reduction in both shear modulus and bulk modulus of the tumor by half relative to their baseline values prior to treatment (Panagi et al., 2024). Simultaneously, the hydraulic conductivity in the model increases linearly by two orders of magnitude compared to its initial value. Detailed numerical values for the hydraulic conductivity k_th_, shear modulus μ, and bulk modulus k, for the host tissue, untreated tumor tissue, and tumor tissue under the influence of ketotifen are provided in Supplementary Table S1 of the SM Appendix.

Although ketotifen is used as a mechanotherapeutic agent in the published experimental dataset that we employed, the model could be similarly adapted to other mechanotherapeutics. This is because the therapeutic mechanism remains the same across different mechanotherapeutic drugs: to decompress blood vessels and enhance perfusion by alleviating mechanical stress and reducing tumor stiffness (Sheridan, 2019). Many mechanotherapeutics—such as antihypertensives, antifibrotics, or antihistamines—are commonly used drugs that are repurposed to normalize the tumor microenvironment. They typically act by targeting extracellular matrix (ECM) components or by reprogramming cancer-associated fibroblasts (CAFs), with the shared goal of restoring vessel function and improving the delivery of therapeutic agents (Mpekris et al., 2024).

#### 2.2.4 Implementation of sonopermeation

Sonopermeation is recognized for its ability to temporarily increase the size of vessel wall pores, thereby enhancing vascular permeability and leading to a marked improvement in the functional density of the vascular network. Notably, experimental studies have shown that sonopermeation can enlarge endothelial cell pores to dimensions ranging from approximately 100 nm to 1.25 μm (Qiu et al., 2012). Furthermore, it has been observed that larger pore sizes are positively associated with either an increase in acoustic pressure or a prolonged duration of sonopermeation treatment (Qiu et al., 2012). Building on the aforementioned experimental findings—particularly the observation that increasing acoustic pressure during sonopermeation leads to the formation of larger vessel pores—we derived a second-order polynomial function based on these experimental data. This function predicts vessel wall pore size, r_o_, as a function of acoustic pressure, p_ac_. The resulting equation (Equation 6) was incorporated into our mathematical model by integrating it into the r_o_ parameter. This allowed for a quantitative description of the relationship between p_ac_ and r_o_, enhancing the model’s ability to accurately represent the underlying biological phenomenon.

$$r_{o}=-14977.9087\cdot p_{\text{ac}}^{2}+8208.3947\cdot p_{\text{ac}}-69.0722,$$ (6)

The incorporation of acoustic pressure into our mathematical model is achieved through the following formulation:

$$p_{\text{ac}}=\text{MI}\cdot \sqrt{\text{fr}}$$ (7)

The acoustic pressure, p_ac_, is mathematically defined as the product of the Mechanical Index (MI) of the transducer and the square root of the frequency, fr, used during sonopermeation for the transmitted wave (Husseini, Pitt, and Martins, 2014; Kooiman et al., 2014). The selection of values of mechanical index and frequency is based on previous clinical and experimental studies (Mpekris et al., 2024; Dimcevski et al., 2016; Wang et al., 2018; Snipstad et al., 2018; Snipstad, Vikedal, et al., 2021). Specifically, based on experimental protocol (Mpekris et al., 2024), in which a clinical ultrasound device was used to perform sonopermeation, optimal MI values in the range of 0.2–0.6 were identified for enhancing drug delivery. Values of frequency and mechanical index are given in SM Appendix, Supplementary Table S1. These values that we use in our model are close to the values which are employed in clinical trials (Dimcevski et al., 2016; Wang et al., 2018). Importantly, in Equations 6, 7, we neglect any attenuation and scattering effects due to the propagation of ultrasound waves and interaction with tissues and thus, the acoustic pressure remains constant and distributed uniformly within the tissue (Blanco et al., 2025; Blanco et al., 2021).

Furthermore, within the framework of our mathematical model, we have incorporated the shear wave stresses, denoted as τ, which are exerted upon endothelial cells as a direct consequence of the microbubbles inserted during the sonopermeation process. The phenomenon of wall shear stress associated with sonopermeation arises from the oscillatory motion of gas microbubbles when subjected to an ultrasonic field that is situated in close proximity to the surface of the tissue. These oscillations of the bubbles are known to induce a steady shear stress, which can be attributed to the mechanism of microstreaming (Krasovitski and Kimmel, 2004). The expression for the shear wave stress is described by Equation 8 (Cowley and McGinty, 2019).

$$\tau =2{\left(\rho_{L}\cdot \mu_{L}\right)}^{\frac{1}{2}}{\left(\pi \cdot \text{fr}\right)}^{\frac{3}{2}}\frac{\eta_{m}^{2}}{R_{0}}$$ (8)

where ρ_L_ represents the density of the liquid medium surrounding the pulsating shelled microbubble and μ_L_ is the viscosity of this surrounding liquid medium. In the current case, the liquid medium is the blood. Moreover, η^2^_m_ is the displacement amplitude of the microbubble wall and R_0_ is the equilibrium radius of the shelled microbubble (Lewin and Bjørnø, 1982; Rooney, 1972).

Experimental evidence has demonstrated that wall shear stress operates as a significant stimulus for both the proliferation and apoptosis of endothelial cells (Dolan et al., 2011). Drawing from the previously discussed experimental findings, which demonstrate that wall shear stress plays a crucial role in regulating both the proliferation and apoptosis of endothelial cells, we extracted the relevant experimental data to develop fitting equations that can be integrated into our mathematical model. Specifically, these fitting equations, which capture how wall shear stress influences both cell proliferation (pr) and apoptosis (ap) are formulated as second-order polynomial functions. These functions serve to describe the relationship between the mechanical stimulus provided by wall shear stress and the cellular responses of proliferation and apoptosis, providing a more accurate representation of these biological processes within the context of our model.

$$\mathrm{pr}=-3\cdot 10^{-6}\cdot \tau^{2}+0.0067\cdot \tau +0.2533$$ (9)

$$\text{ap}=-3\cdot 10^{-5}\cdot \tau^{2}+0.0126\cdot \tau +0.6666$$ (10)

The Equations 9, 10 are to be multiplied by the proliferation and apoptosis terms associated with the endothelial cells, which can be found on the right-hand side of the transport equation for endothelial cells, (Equation S29, SM Appendix).

#### 2.2.5 Solution of model equations

To accurately simulate tumor growth, the tumor is assumed to have a spherical geometry, surrounded by a cubic domain representing normal tissue. The cubic host domain is designed to be two orders of magnitude larger than the tumor to minimize boundary effects that could influence tumor progression. Owing to the symmetry inherent in the system, the analysis is confined to one-eighth of the entire domain, which is sufficient for capturing the system’s behavior (Mpekris, Baish, et al., 2017; Mpekris et al., 2020; Harkos and Stylianopoulos, 2024; Harkos, Stylianopoulos, and Jain, 2023; Kim, Stolarska, and Othmer, 2011).

The mathematical model’s system of equations was implemented and solved using the commercial finite element software COMSOL Multiphysics (COMSOL, Inc., Burlington, MA, United States). The simulation employed the Solid Mechanics, Transport of Diluted Species, Convection-Diffusion Equation, and Domain ODEs and DAEs physics interfaces. The computational domain comprised 6,015 finite elements and 51,628 degrees of freedom. A time-dependent solver, specifically the PARDISO algorithm, was used to obtain solutions to the governing equations.

Details regarding the boundary conditions utilized in this study are provided in the SM Appendix, Supplementary Figure S1. At the interface between tumor and healthy tissue, boundary conditions governing stress and displacement fields, as well as the concentrations of oxygen and immuno-nanotherapeutic agents, were automatically applied by the software. In COMSOL Multiphysics, such continuity conditions at internal boundaries are enforced when adjacent domains are governed by the same physics interface to ensure continuity of the relevant field variables and their fluxes across internal interfaces.

## 3 Results

### 3.1 Comparison of model predictions with experimental data

To assess the accuracy of our mathematical framework and to justify the parameter values utilized within the model, we compared model predictions with previously published experimental data (Neophytou et al., 2025). The data were derived from *in vivo* experiments conducted on murine breast tumor models, which aimed to identify optimal conditions for combining mechanotherapeutics with ultrasound sonopermeation to enhance perfusion and the efficacy of nano-immunotherapy (Neophytou et al., 2025). The experimental findings demonstrated that the use of ketotifen, an anti-histamine with mechanotherapeutic properties, in conjunction with sonopermeation led to a significant reduction in tumor stiffness within the TME. This reduction was attributed to a decrease in collagen and hyaluronan levels, effectively remodeling the TME. The synergistic effects of ketotifen and sonopermeation resulted in a remarkable increase in tumor perfusion and a substantial enhancement in drug delivery. As a result of these improvements, the therapeutic efficacy of both Doxil nanomedicine and ICI cocktail (anti-PD-1 and anti-CTLA-4) was significantly elevated, highlighting the potential of this combined approach to optimize treatment outcomes in a tumor setting. These findings underscore the effectiveness of integrating mechanotherapeutics with sonopermeation to overcome physical barriers in the TME and improve therapeutic delivery and efficacy.

**FIGURE 3:**
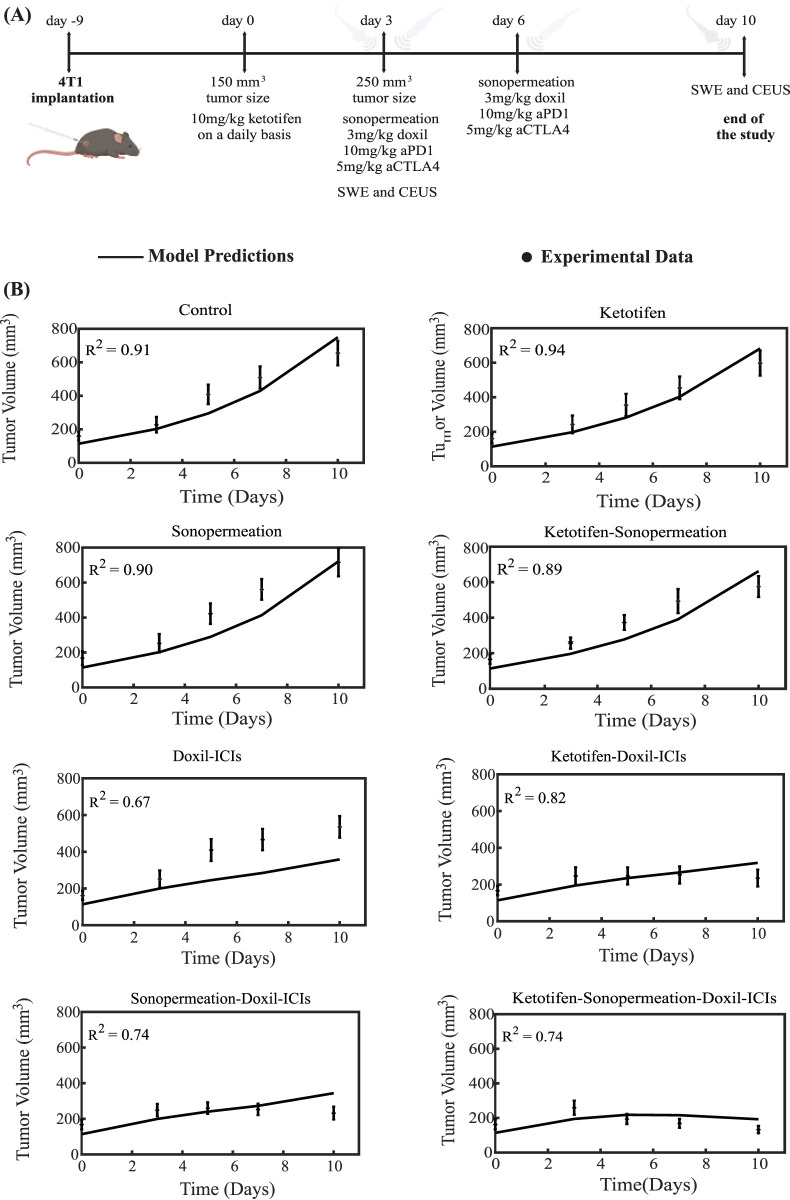
Comparison of model predictions with experimental data of tumor growth for 4T1 breast tumors. **(A)** Experimental treatment protocol implemented for 4T1 breast tumors, which was also simulated using the mathematical model. Created in BioRender.com. **(B)** Tumor volume growth rates of murine breast cancer cells are presented, with experimental data represented by dots and mathematical model predictions depicted as solid lines for each treatment group. For each case - control, ketotifen, sonopermeation, ketotifen-sonopermeation, Doxil-ICIs, ketotifen-Doxil-ICIs, sonopermeation-Doxil-ICIs and ketotifen-sonopermeation-Doxil-ICIs- the R-Squared (R^2^) value has been calculated and depicts the accuracy of mathematical model validations for tumor growth in comparison with experimental findings. ICIs denotes cocktail of anti-PD1 and anti-CTLA4 antibodies.

**FIGURE 4:**
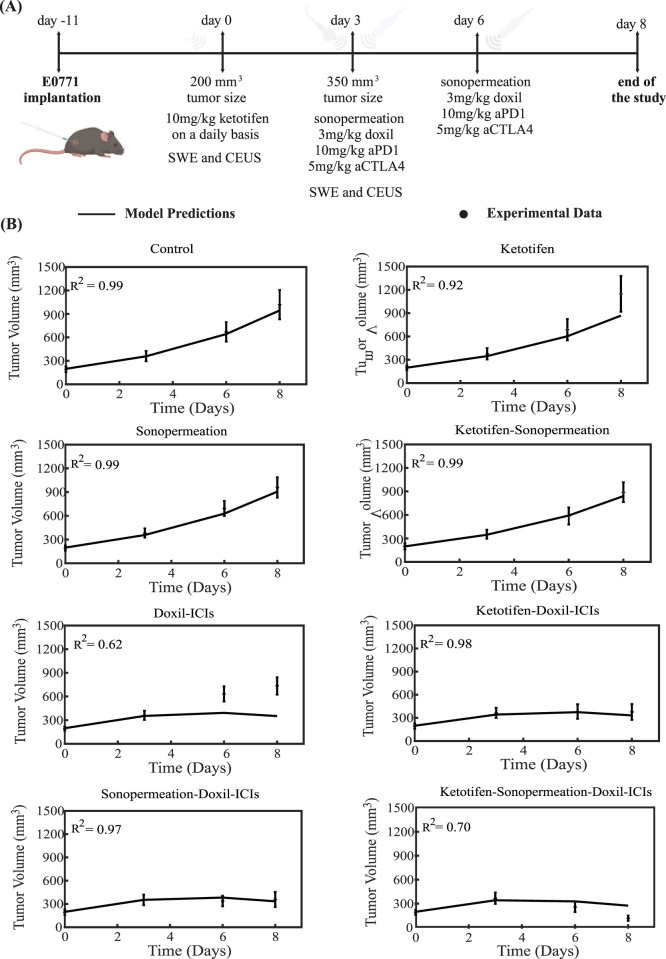
Comparison of model predictions with experimental data of tumor growth for E0771 breast tumors. **(A)** Experimental treatment protocol implemented for E0771 breast tumors, which was also simulated using the mathematical model. Created with BioRender.com. **(B)** Tumor volume growth rates of murine breast cancer cells are presented, with experimental data represented by dots and mathematical model predictions depicted as solid lines for each treatment group. For each case - control, ketotifen, sonopermeation, ketotifen-sonopermeation, Doxil-ICIs, ketotifen-Doxil-ICIs, sonopermeation-Doxil-ICIs and ketotifen-sonopermeation-Doxil-ICIs- the R-Squared (R^2^) value has been calculated and depicts the accuracy of mathematical model validations for tumor growth in comparison with experimental findings. ICIs denotes cocktail of anti-PD1 and anti-CTLA4 antibodies.

The therapeutic regimen simulated through the mathematical model was designed to closely replicate the experimental protocol applied to 4T1 and E0771 breast tumors, as depicted in Figures 3A, 4A. For the experimental protocol, mice were randomized into the following groups once tumors reached an average size of 150–200 mm^3^ (n = 8–10 per group): Control group, ketotifen (10 mg/kg, i. p.), sonopermeation, ketotifen + sonopermeation, Doxil (3 mg/kg, i. v.) + immune checkpoint inhibitors (ICIs; a cocktail of anti-PD-1, 10 mg/kg, and anti-CTLA-4, 5 mg/kg, i. p.), ketotifen + Doxil + ICIs, sonopermeation + Doxil + ICIs, and ketotifen + sonopermeation + Doxil + ICIs. Daily administration of ketotifen began once tumors reached approximately 150 mm^3^. After 3 days of ketotifen treatment—by which time tumors had grown to an average volume of 250–350 mm^3^—mice were subjected to sonopermeation. One hour later, Doxil and ICIs were administered to enhance therapeutic efficacy. The combined therapy involving sonopermeation and nano-immunotherapy was repeated 3 days later (Neophytou et al., 2025). Throughout the experimental process, tumor volume was measured using a digital caliper, while tumor elastic modulus and perfused area were evaluated on specific days using Shear Wave Elastography (SWE) and Contrast-Enhanced Ultrasound (CEUS), respectively.

To compare the model’s predictions with the experimental data, all parameters in the model were assigned baseline values independently derived from prior studies (SM Appendix, Supplementary Table S1). The sole parameter modified to align the model’s predictions with the experimental observations (Neophytou et al., 2025) was the parameter denoted as k_1_, which quantifies the relationship between cancer cell proliferation and oxygen concentration (SM Appendix, Equation S8). The value of k_1_ was adjusted to ensure that the predicted final tumor volume for the untreated control group matched the experimental measurement. This value remained consistent across all comparisons between model’s predictions and the experimental data for the various groups studied. Remarkably, despite the mechanistic complexity of the model and the considerable number of parameters it incorporates, only a single parameter -*k*_*1*_- was varied for each breast cancer type to achieve alignment between the model predictions and the experimental results (SM Appendix, Supplementary Table S2). This parameter (*k*_*1*_) governs the proliferation rate of cancer cells, which varies across different cancer cell lines, and it was fitted so that model predictions reproduce the data of the control (untreated) groups in the experiments. Since each cell line used in the experimental data exhibits a distinct growth profile, calibrating *k*_*1*_ for each type ensures that the model accurately reflects the biological variability in tumor growth rates.

The tumor volume predictions generated by the mathematical model align closely with experimental findings. The results show that monotherapies—including the control group, ketotifen, sonopermeation, and the combination of ketotifen with sonopermeation—failed to exhibit notable antitumor effects, as no notable reductions in tumor volume were observed when compared to the control group. However, a marked reduction in tumor volume was evident with the application of the combinatorial treatment. The model confirms that integrating ketotifen and sonopermeation with nano-immunotherapy notably enhances therapeutic efficacy, particularly by suppressing tumor growth (Figures 3B, 4B). The most pronounced delays in tumor progression for both 4T1 and E0771 breast tumor models were observed when the treatment protocol combined ketotifen, sonopermeation, Doxil, and immune checkpoint inhibitors (ICIs), specifically anti-PD-1 and anti-CTLA-4 antibodies. This combination demonstrated a substantial reduction in tumor volume, achieving effective therapeutic outcomes. The accuracy of the mathematical model in replicating experimental results can be assessed through the R-Squared (R^2^) statistic. This metric evaluates the degree of correlation between the experimental data and the model’s predictions, with R^2^ values ranging from 0 to 1. A higher R^2^ value, approaching 1, signifies stronger precision and reliability of the model’s predictive capabilities.

**FIGURE 5:**
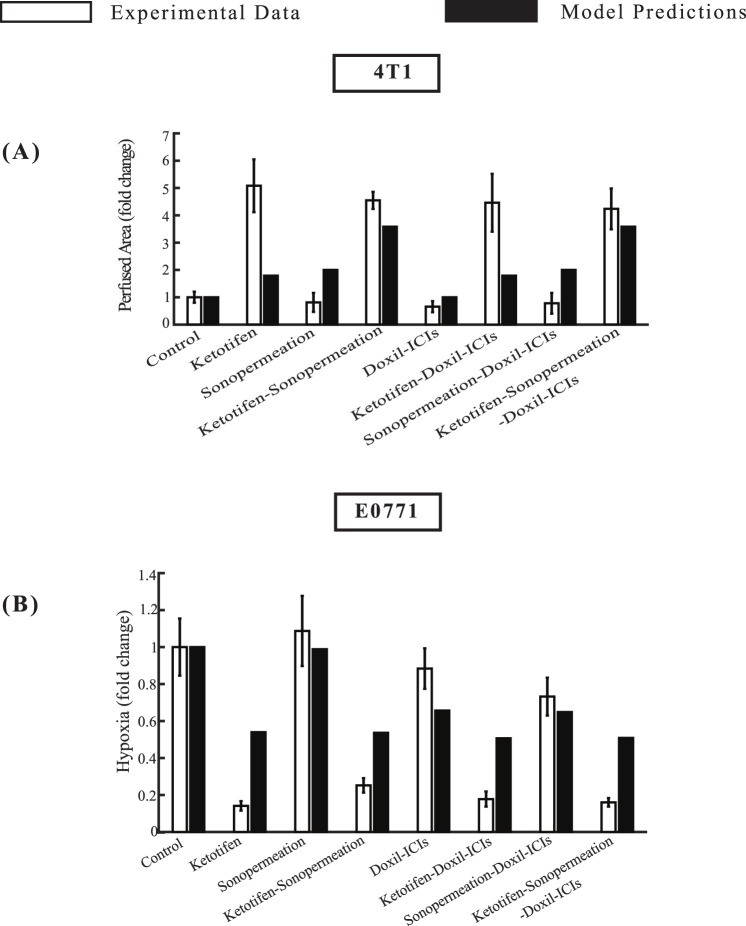
Comparative of model predictions alongside experimental data (Neophytou et al., 2025) at a specific time point. The horizontal axis represents the various treatment groups that were examined in the experimental investigations: control, ketotifen, sonopermeation, ketotifen-sonopermeation, Doxil-ICIs, ketotifen-Doxil-ICIs, sonopermeation-Doxil-ICIs, ketotifen-sonopermeation-Doxil-ICIs. The vertical axis (y) for each instance varies between **(A)** Perfused Area for 4T1 breast tumors, **(B)** Hypoxia for E0771 breast tumors.

To provide a detailed comparison between mathematical model predictions and experimental data, we analyzed model outcomes alongside experimental measurements of perfused area evaluated with CEUS, and hypoxia levels measured with immunofluorescence staining. The results are presented in Figure 5, where the values of measured parameters are expressed relative to those of the control group, shown as fold changes. This approach facilitates direct correlation between the dimensionless parameters of the model and the experimental observations. The computational data for the perfused area within the tumor was quantified and analyzed through our mathematical framework by calculating the functional vascular density, Sv, as outlined in Equation S27, SM Appendix. In our mathematical model, hypoxia levels were assessed by calculating the oxygen concentration within the tumor tissue. To create a relative metric for comparison, the oxygen concentration in the tumor was divided by the oxygen concentration in healthy tissue, which is maintained at a stable value of 0.2 mol/m^3^. This ratio provided a quantitative measure of the reduction in oxygen levels within the tumor relative to normal conditions. To further refine this measure, the ratio was converted into a percentage representing the oxygen concentration in the tumor. The complementary percentage was then defined as the level of hypoxia, offering a clear representation of the extent to which the tumor environment was deprived of oxygen. To facilitate a meaningful comparison between the computational predictions and experimental data, the computed results were normalized and expressed as fold changes. This transformation ensured that the predictions from the model could be directly compared to the experimental findings, thereby enhancing the evaluation process.

The model predictions align closely with experimental data regarding perfused area, and hypoxia levels within the TME, as depicted in Figure 5. When ketotifen is combined with sonopermeation or used as part of the comprehensive treatment regimen involving ketotifen, sonopermeation, Doxil, and immune checkpoint inhibitors (ICIs), there is a notable enhancement in the perfused area, contributing to improved functional vascular density (Figure 5A). A comparison of hypoxia levels across various experimental groups reveals that model predictions also agree well with experimental findings. The administration of ketotifen substantially reduces hypoxia levels within the TME (Figure 5B). This reduction in hypoxia is particularly evident in treatment modalities that combine ketotifen, sonopermeation, and nano-immunotherapy, highlighting the synergistic effects of these interventions. By mitigating hypoxia, these therapies effectively enhance drug delivery, further validating the therapeutic potential of the combinatorial approach.

### 3.2 Parametric analysis of key model variables

The aim of the parametric analysis, which systematically assessed various parameters, was to develop essential guidelines for optimizing the sequence of experimental procedures and determining the ideal time intervals between their application to achieve the best therapeutic outcomes. Specifically, the analysis explored two distinct scenarios: one in which sonopermeation is administered prior to nano-immunotherapy, and another where nano-immunotherapy precedes ultrasound sonopermeation. In the experimental setup, sonopermeation was applied first, followed by nano-immunotherapy 1 hour later (Mpekris et al., 2024; Neophytou et al., 2025). In the first scenario, where the effect of sonopermeation precedes nano-immunotherapy, sonopermeation was applied at two fixed time intervals. Particularly, the first sonopermeation’s effect was implemented on day 19, followed by a second treatment on day 22. To evaluate the effectiveness of nano-immunotherapy in this sequence, its timing was varied, and it was delivered at four different intervals: 1 h, 3 h, 6 h, and 24 h after the initiation of sonopermeation. In the second scenario, where nano-immunotherapy was administered before sonopermeation, the nano-immunotherapy treatment was also delivered at two fixed time points, on day 19 and day 22. In this case, the effect of ultrasound sonopermeation was executed at varying time intervals relative to the administration of nano-immunotherapy, specifically at 1 h, 3 h, 6 h, and 24 h afterward. Based on these parameters, the procedures were integrated and simulated within a mathematical model to identify the optimal sequence and timing for achieving the desired therapeutic outcomes in this combined treatment approach.

**FIGURE 6:**
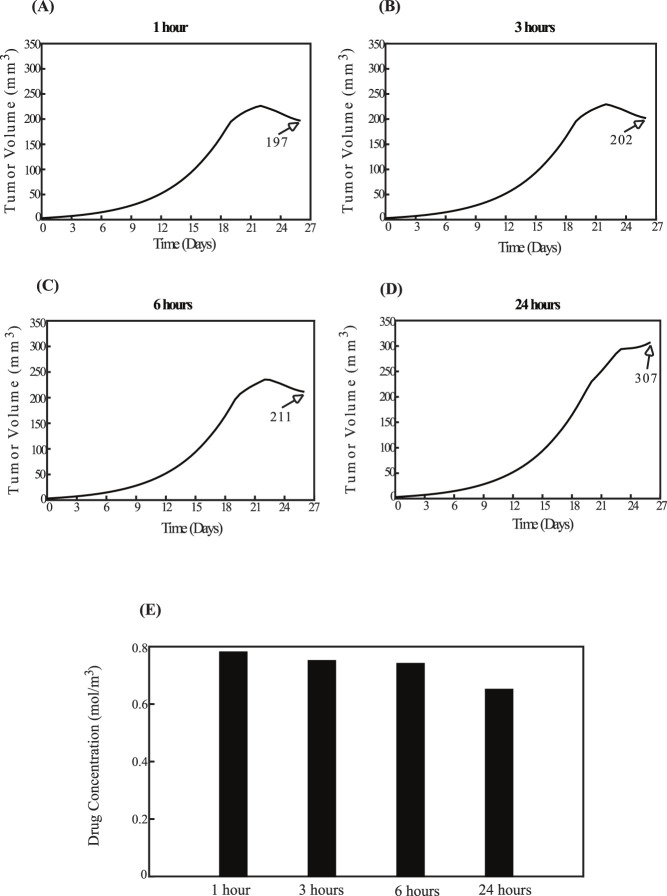
Effect of the order of therapeutic modality application. The impact of administering sonopermeation prior to nano-immunotherapy. The effect of sonopermeation is applied at fixed time intervals, while nano-immunotherapy is introduced at varying intervals—1 h, 3 h, 6 h, and 24 h—following ultrasound sonopermeation. Specifically, panel **(A)** corresponds to a 1-h interval, **(B)** to a 3-h interval, **(C)** to a 6-h interval, and **(D)** to a 24-h interval between sonopermeation and nano-immunotherapy. The diagrams illustrate the effects of the elapsed time on tumor volume (mm^3^) in panels **(A–D)**, as well as on drug concentration in panel **(E)**.

Figure 6 illustrates the effect of administering sonopermeation prior to nano-immunotherapy. The smallest tumor volume is observed in Figure 6A, where nano-immunotherapy is applied 1 h after sonopermeation. Conversely, the largest tumor volume is seen in Figure 6D, where nano-immunotherapy is administered 24 h after the application of sonopermeation. Intermediate time intervals of 3 h and 6 h, shown in Figures 6B,C, respectively, do not exhibit a pronounced difference when compared to Figure 6A. This observation can be supported by existing evidence, which indicates that the primary effect of sonopermeation typically persist for a duration of approximately up to 6 h (Sheikov et al., 2008; Yuana et al., 2017; Zhang et al., 2009; Poon, McMahon, and Hynynen, 2017). Furthermore, these observations are corroborated by the drug concentration within the TME, as depicted in Figure 6E. The drug concentration is the highest when nano-immunotherapy is administered 1 hour after sonopermeation and the lowest when administered 24 h after sonopermeation.

**FIGURE 7:**
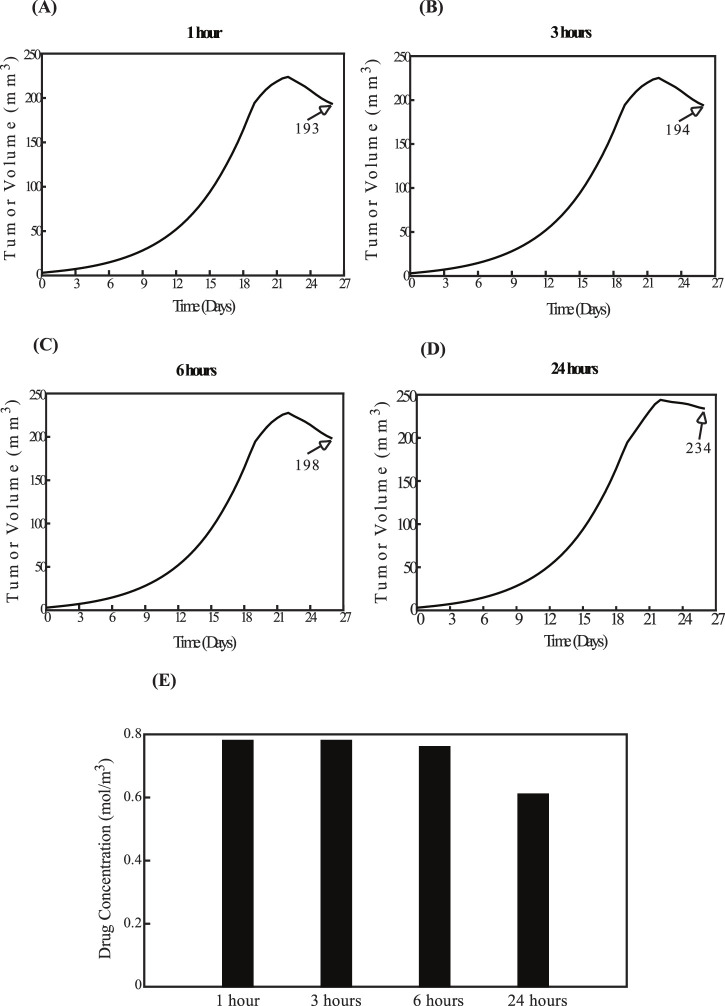
Effect of the order of therapeutic modality application. The impact of administering nano-immunotherapy prior to sonopermeation. The administration of nano-immunotherapy is applied at fixed time intervals, while the effect of sonopermeation is applied at varying intervals—1 h, 3 h, 6 h, and 24 h—following nano-immunotherapy. Specifically, panel **(A)** corresponds to a 1-h interval, **(B)** to a 3-h interval, **(C)** to a 6-h interval, and **(D)** to a 24-h interval between nano-immunotherapy and sonopermeation. The diagrams illustrate the effects of the elapsed time on tumor volume (mm^3^) in panels **(A–D)**, as well as on drug concentration in panel **(E)**.

Figure 7 examines the reverse order, with nano-immunotherapy preceding sonopermeation. The findings align with those in Figure 6. Specifically, the smallest tumor volume occurs when the impact of sonopermeation is applied 1 hour after nano-immunotherapy, as shown in Figure 7A, while the largest tumor volume is observed when sonopermeation is administered 24 h after nano-immunotherapy, as shown in Figure 7D. These results are further validated by drug concentration data (Figure 7E). For intermediate time intervals of 3 h and 6 h, shown in Figures 7B,C, no notable differences are observed compared to Figure 7A.

A comparison of Figures 6, 7 reveals that for time intervals of 1 h, 3 h, and 6 h (Figures 6A–C; Figures 7A–C), the treatment schedule—whether the impact of sonopermeation or nano-immunotherapy is applied first—does not sufficiently influence tumor volume. However, at the 24-h interval, the schedule becomes critical. The parametric analysis indicates that administering nano-immunotherapy first, followed by the effect of ultrasound sonopermeation, results in a smaller tumor volume (Figure 7D) compared to the reverse sequence, where the impact of sonopermeation precedes nano-immunotherapy (Figure 6D), which leads to a notably larger tumor volume.

## 4 Discussion

In this study, we developed a mechanistic mathematical framework designed to integrate the synergistic effects of mechanotherapeutics and sonopermeation, with the goal of maximizing the therapeutic efficacy of nano-immunotherapy in cancer treatment. The proposed model captures the intricate interactions among a wide range of cellular and molecular components within the TME, including various types of cancer cells, immune cells, tumor-associated macrophages, endothelial cells, tumor angiogenic factors, and multiple therapeutic modalities, each contributing distinct and significant effects. Our framework represents a substantial advancement over previous studies by incorporating the dual effects of sonopermeation and the mechanotherapeutic agent ketotifen, which has demonstrated promising therapeutic potential. The robustness of the model is evidenced by its ability to predict outcomes that align closely with experimental results derived from *in vivo* studies on two breast cancer cell lines: the E0771 and 4T1 mammary adenocarcinoma cell lines. The experimental data collected strongly support the notion that mechano-modulation of the TME, achieved through the combined application of mechanotherapeutics and sonopermeation, induces synergistic effects that significantly enhance perfusion and improve overall therapeutic outcomes (Neophytou et al., 2025). This observation is further corroborated by the mathematical model, which demonstrates a high degree of precision in replicating experimental results. Specifically, a strong correlation was observed between the model’s predictions and actual experimental measurements of key parameters, including tumor volume, functional vascular density, and hypoxia levels. These correlations underscore the accuracy and reliability of the proposed mathematical framework, reinforcing its potential as a powerful tool for optimizing cancer therapies.

Beyond breast cancer (Neophytou et al., 2025), the proposed model can also be readily adapted and validated for other tumor types, such as pancreatic cancer (Murphy et al., 2019) and sarcomas (Mpekris et al., 2024). These tumors share key characteristics, including a dense extracellular matrix which limits drug penetration and perfusion—making them suitable targets for mechano-modulatory strategies in combination with sonopermeation. The primary variable that differs across these desmoplastic tumors is the growth rate. In our mathematical framework, the primary tumor-specific parameter is the cancer cell proliferation rate constant (*k*_*1*_), which governs the tumor growth rate. By adjusting this parameter, the model can be calibrated to match experimental data from various tumor types without requiring other changes to the model itself. This flexibility underscores the model’s generalizability and supports its potential utility across a broad spectrum of solid tumors characterized by a stiff and poorly perfused microenvironment.

The parametric analysis, which systematically evaluated a range of parameters, established critical guidelines for optimizing the sequence of experimental procedures. It also identified the ideal time interval required between the application of sonopermeation and the commencement of the combined Doxil + anti-PD-1+anti-CTLA-4 treatment. Specifically, the analysis distinguishes two scenarios: one where sonopermeation precedes nano-immunotherapy, as is also reflected in the experimental protocol (Neophytou et al., 2025), and another where nano-immunotherapy is administered prior to ultrasound sonopermeation. Moreover, it determines the optimal interval between these two therapeutic modalities. This analysis provides key insights, ensuring the most favorable and effective outcomes for this combined treatment strategy.

While the combined use of mechanotherapeutics and sonopermeation to enhance nano-immunotherapy has not yet been investigated in the clinic, each component independently shows significant promise. Losartan has helped establish the term “mechanotherapeutics” as a novel therapeutic strategy, owing to its early successes in clinical trials. In patients with locally advanced pancreatic cancer, treatment with FOLFIRINOX and losartan followed by chemoradiotherapy was associated with an increased R0 resection rate—an outcome linked to prolonged progression-free and overall survival (Murphy et al., 2019). The mechanotherapeutic agent ketotifen has been shown to reduce tumor stiffness and improve perfusion in sarcoma models, thereby enhancing the efficacy of both immunotherapy and nanomedicines (Charalambous et al., 2024; Panagi et al., 2024). Importantly, this effect is being evaluated in sarcoma patients, with ongoing clinical evaluation (EudraCT Number: 2022-002311-39), underscoring ketotifen’s translational potential (Panagi et al., 2024). Separately, clinical studies using ultrasound and microbubbles have demonstrated enhanced chemotherapy response, prolonged survival in patients with pancreatic ductal adenocarcinoma (Dimcevski et al., 2016), and increased chemosensitivity in gastrointestinal malignancies (Wang et al., 2018). Together, these findings support the clinical relevance of our modeling framework and highlight the promise of this combined strategy for future therapeutic development.

The model presents certain limitations. First, due to the inherent symmetry of the system, the analysis is limited to one-eighth of the full domain, which is sufficient to capture the system’s behavior. Moreover, our mathematical model does not simulate the spatio-temporal propagation of ultrasound waves. As a result, it does not account for physical phenomena such as the dynamics of acoustic energy delivery, including attenuation and scattering. Instead, the model focuses on representing the biological effects of sonopermeation on various components of the tumor microenvironment, as informed by experimental observations. Additionally, in modeling tumor growth, the model does not capture the high-frequency oscillations and accelerations associated with ultrasound at the microsecond timescale—a duration much shorter than the timescale relevant for tumor growth (Blanco et al., 2025). The model also does not incorporate the effects of sonopermeation on intracellular signaling pathways, such as the phosphorylation of MAP-kinases (MAPK) (Haugse et al., 2020), or the activation of p38, ERK, Akt, and integrin receptors, including focal adhesion kinase (FAK) (Whitney et al., 2012; Takeuchi et al., 2008; Sato et al., 2014; Przystupski and Ussowicz, 2022). These limitations are expected to affect the quantitative accuracy of the model’s predictions. However, the core conclusions drawn from this study are not expected to be impacted by these constraints.

## Data Availability

The datasets analyzed for this study can be found in the Zenodo, 629 https://zenodo.org/records/14531590.
