# Supplementary material for: Synergistic mechanotherapy and sonopermeation guided by mathematical modeling for solid tumor treatment
Source: Front Drug Deliv. 2025 Jun 24;5:1549098. doi: 10.3389/fddev.2025.1549098 (PMC12360438; doi:10.3389/fddev.2025.1549098)
Supplement: Supplementary file 1 [file DataSheet1.pdf]

Supplementary Material

Synergistic Mechanotherapy and Sonopermeation Guided by Mathematical Modeling for Solid Tumor Treatment

Marina Koutsi, Triantafyllos Stylianopoulos, Fotios Mpekris

1 Description of the Mathematical Model

Kinematic principles underlying tumor growth

Equations (S1-S3) and (S18-S26) establish the comprehensive framework for the biomechanical model of tumor growth, which is solved to simulate the progression of the tumor within normal/host tissue. The solutions to these equations facilitate the computation of interstitial fluid pressure, fluid velocity, and the stresses generated through mechanical interactions between the tumor and the host tissue. Interstitial fluid pressure and fluid velocity are incorporated into the drug transport equations (Equations 1-3, 4, in the main text), while the mechanical interactions between the tumor and the host tissue influence the tumor's growth rate. Equations (S4-S17) serve to clarify the balance law concerning the various cell types within the tumor to measure the growth stretch ratio, λ_g, which determines the rate of tumor proliferation. This growth stretch ratio is utilized within the general framework via Equations (S2, S3, and S25). All parameter values of this mathematical model are detailed in **Supplementary Table 1**. This mathematical framework employs a robust systems-biology approach, which is meticulously designed to incorporate the intricate cellular and subcellular phenomena that occur within biological systems, specifically utilizing a continuum mechanics and finite elements model to thoroughly analyze the multifaceted dynamics of tumor growth.

In order to describe the kinematic behavior of the tumor, the multiplicative decomposition of the deformation gradient tensor is utilized. The tensor $\mathbf{F}$ is divided into two distinct components: the tumor's growth, denoted as $\mathbf{F_g}$, and the elastic mechanical interaction, represented as $\mathbf{F_e}$ (Ambrosi and Mollica 2002; Mpekris et al. 2015; Rodriguez, Hoger, and McCulloch 1994; Skalak et al. 1996):

$$\mathbf{F} = \mathbf{F_e}\mathbf{F_g} \tag{S1}$$

Henceforth, all tensor representations will be rendered in **boldface** to facilitate their distinction from other terms. It is important to recognize that, given the assumptions inherent in the model and the specific problem under investigation, the deformation gradient tensor can be expressed as a combination of more than two independent components, which separately encompass growth, residual stress formation, or extracellular matrix (ECM) remodeling (Skalak et al. 1996; Stylianopoulos and Jain 2013; Mascheroni et al. 2018). The growth component, denoted as $\mathbf{F_g}$, has been characterized as isotropic and homogeneous, representing a non-stress inducing deformation gradient tensor that represents the proliferation of cancer cells (Kim, Stolarska, and Othmer 2011; Roose et al. 2003; Stylianopoulos et al. 2013; Pirentis et al. 2015).

$$\mathbf{F_g} = \lambda_g \mathbf{I} \tag{S2}$$

where λ_g is the growth stretch ratio, which assesses the proliferation of cancer cells, while $\mathbf{I}$ represents the second-order identity tensor, indicating that the proliferation associated with tumor growth occurs uniformly and isotropically. The elastic component, $\mathbf{F_e}$, accounts for the stress-inducing elastic interactions both within the tumor and with the surrounding host tissue. The elastic component of $\mathbf{F}$ was derived directly from Equation (S1) as:

$$\mathbf{F_e} = \mathbf{F}\mathbf{F}_g^{-1} \tag{S3}$$

Calculation of the growth stretch ratio λ_g

The rate of tumor growth is measured by taking into account both the concentration of oxygen and the proliferation of cancer cells (Voutouri and Stylianopoulos 2014; Kim, Stolarska, and Othmer 2011; MacLaurin et al. 2012; Mpekris et al. 2018; Roose et al. 2003). The equation utilized is:

$$\frac{d\lambda_g}{dt} = \frac{1}{3}\left(\frac{T_{CCs}}{T_{tot}} S_{CCs} + \frac{T_{SCCs}}{T_{tot}} S_{SCCs} + \frac{T_{ICCs}}{T_{tot}} S_{ICCs}\right)\lambda_g, \tag{S4}$$

where T_{CCs} represents the population of non-stem cancer cells (CCs), T_{SCCs} denotes the stem cancer cells (SCCs) population, T_{ICCs} signifies the induced cancer cells (ICCs) population, T_{tot} constitutes the total density of tumor cells as determined by the summation of the aforementioned three populations. The S_{CCs}, S_{SCCs} and S_{ICCs} are the respective rates of proliferation and degradation for the CCs, SCCs and ICCs, as specified subsequently in Equations (S9-S11).

Components of the tumor microenvironment

As outlined in the current mathematical framework, we incorporate the interactions that occur among cancer cells, immune system cells, and tumor-associated macrophages, which will be elaborated upon subsequently.

Cancer Cells

The dynamics of the populations of non-stem cancer cells (CCs), stem cancer cells (SCCs), and induced cancer cells (ICCs) are delineated by Equations (S5-S7). SCCs exhibit resistance to pharmacotherapeutic agents, hypoxic conditions, and the immune response, whereas ICCs, upon the administration of nano-immunotherapy, adopt a more stem-like phenotype.

$$\frac{\partial T_{CCs}}{\partial t} = \nabla \cdot (D_{CCs} \nabla T_{CCs}) + GS_f T_{CCs} - cNT_{CCs} - D + p_{CT} T_{SCCs} + p_{IT} T_{ICCs} - (p_{TC} + p_{TI}) T_{CCs} - \lambda_{M1} M_1 T_{CCs} \tag{S5}$$

$$\frac{\partial T_{SCCs}}{\partial t} = \nabla \cdot (D_{SCCs} \nabla T_{SCCs}) + \alpha_{SCCs} G S_{f_{SCCs}} T_{SCCs} - c_{SCCs} N C_{SCCs} - D_{SCCs} + p_{TC} T_{CCs} + p_{IC} T_{ICCs} - (p_{CT} + p_{CI}) T_{SCCs} \quad \text{(S6)}$$

$$\frac{\partial T_{ICCs}}{\partial t} = \nabla \cdot (D_{ICCs} \nabla T_{ICCs}) + \alpha_{ICCs} G S_{f_{ICCs}} T_{ICCs} - c_{ICCs} N T_{ICCs} - D_{ICCs} + p_{TI} T_{CCs} + p_{CI} T_{SCCs} - (p_{IT} + p_{IC}) T_{ICCs} \quad \text{(S7)}$$

In this context, N represents the Natural Killer (NK) cells, M_1 corresponds to the M_1 tumor-associated macrophages (TAMs), while D_{CCs}, D_{SCCs}, and D_{ICCs} denote the diffusion coefficients of non-stem, stem, and induced cancer cells, respectively. To incorporate the influence of drug delivery on growth, the surviving fractions of cancer cells, denoted as S_f, $S_{f_{SCCs}}$, and $S_{f_{ICCs}}$, are integrated into Equations (S5), (S6), and (S7), respectively. The variables c, c_{SCCs}, c_{ICCs} and D, D_{SCCs}, D_{ICCs} quantify the respective proportions of tumor cells that are eliminated by NK and $CD8^+$ T-cells. Especially, for the parameters c_{SCCs}, D_{SCCs}, c_{ICCs} and D_{ICCs}, which characterize the cytotoxic potential of immune cells against SCCs and ICCs, we argue that these cells display heightened resistance during interactions with immune entities. Based on empirical evidence (Todaro et al. 2009), the cytotoxic efficacy of $CD8^+$ T-cells against stem cancer cells (SCCs) is considered to be seven times lower than that against CCs. Therefore, the parameters that govern the elimination of SCCs by immune cells are assumed to be the same as for CCs but multiplied by a factor of 0.14.

G characterizes the proliferation of CCs, SCCs, and ICCs as a function of oxygen availability. Regarding the coefficients of the proliferation rates for SCCs and ICCs, namely α_{SCCs} and α_{ICCs}, we claim that under normal oxygen conditions, these values are equivalent to one, thereby ensuring that all cancer cell types exhibit proliferation rates consistent with those of CCs. Conversely, under hypoxic conditions, the proliferation rates of cancer cells exhibiting a stem-like phenotype are observed to increase. Consequently, we hypothesize that their proliferation rates are inversely proportional to the oxygen concentration, such that as the oxygen concentration approaches zero, the proliferation rates double compared to those observed under normal oxygen levels (Conley et al. 2012). The transition rates of cancer cells from type i to type j are denoted by p_{ij} (Goldman et al. 2015). Moreover, the parameter λ_{M1} signifies the tumoricidal activity of M1-like TAMs against cancer cells (Mahlbacher et al. 2018).

The relationship between tumor cells proliferation and the local oxygen concentration, G, is asserted to follow Michaelis-Menten kinetics, taking the form presented in Equation (S8) (Casciari, Sotirchos, and Sutherland 1992a, 1992b):

$$G = \frac{k_1 c_{ox}}{k_2 + c_{ox}} \quad \text{(S8)}$$

where k_1 and k_2 are parameters indicative of the growth rate, and c_{ox} represents the concentration of oxygen.

The mechanism underlying the creation and degradation of the solid phases, S_{CCs}, S_{SCCs} and S_{ICCs} is delineated as follows Equations (S9-S11):

$$S_{CCs} = GS_f T_{CCs} - cNT_{CCs} - D + p_{CT}T_{SCCs} + p_{IT}T_{ICCs} - (p_{TC} + p_{TI})T_{CCs} - \lambda_{M1}M_1T_{CCs} \quad (S9)$$

$$S_{SCCs} = \alpha_{SCCs}GS_{f_{SCCs}}T_{SCCs} - c_{SCCs}NC_{SCCs} - D_{SCCs} + p_{TC}T_{CCs} + p_{IC}T_{ICCs} - (p_{CT} + p_{CI})T_{SCCs} \quad (S10)$$

$$S_{ICCs} = \alpha_{ICCs}GS_{f_{ICCs}}T_{ICCs} - c_{ICCs}NT_{ICCs} - D_{ICCs} + p_{TI}T_{CCs} + p_{CI}T_{SCCs} - (p_{IT} + p_{IC})T_{ICCs} \quad (S11)$$

Immune Cells

For the immune system, this model considers four principal categories of immune cells: natural killer (NK) cells, CD8$^+$ T-cells, CD4$^+$ T-cells and regulatory T-cell (Treg) subset. Utilizing relevant research work (Burroughs et al. 2011; de Pillis, Radunskaya, and Wiseman 2005; Fouchet and Regoes 2008), the system of equations incorporates the recruitment rates of these immune cells, their inactivation by tumor cells, the suppressive function of Tregs and M2 tumor-associated macrophages (TAMs), as well as their mortality rate and interactions with cancer cells. The following Equations (S12-S15) illustrate the interactions that taking place among the cellular elements of the immune system:

$$\frac{\partial N}{\partial t} = \sigma_{nk} - f_{NK}N + \frac{g_{NK}T_{CCs}^{\ 2}}{h + T_{CCs}^{\ 2}}N - p_{im}NT_{CCs} - \lambda_{reg}T_{reg}N - \lambda_{M2}M_2N \quad (S12)$$

$$\frac{\partial L}{\partial t} = \sigma_{T8} - m_{T8}L + \frac{j_{T8}D^2}{k_{im} + D^2}L - qLT_{CCs} + (r_N N + r_{Cd4}C_{d4})T_{CCs} - \lambda_{reg}T_{reg}L - \lambda_{M2}M_2L \quad (S13)$$

$$\frac{\partial C_{d4}}{\partial t} = s_{CD4} + re_{Cd4}C_{d4}\left(1 - \frac{C_{d4}}{C_{d4,max}}\right) - \mu_{C_{d4}}C_{d4} \quad (S14)$$

$$\frac{\partial T_{reg}}{\partial t} = g_{reg}T_{reg} - m_{reg}T_{reg} \quad (S15)$$

where N is the population of NK cells, L of CD8$^+$ T-cells, C_{d4} denotes the population of CD4$^+$ T-cells and T_{reg} represents the population of regulatory T-cells. Additionally, f_{NK}, m_{T8}, and m_{reg} signify the mortality rates of NK cells, CD8$^+$ T-cells, and Treg cells, respectively, while g_{NK}, j_{T8}, and g_{reg} denote the recruitment rates of immune cells, and p_{im} and q represent the inactivation rates of immune cells by cancer cells (CCs). The constants σ_{nk} and σ_{T8} denote the stable sources of NK and CD8$^+$ T-cells, respectively, r_N indicates the rate at which tumor-specific CD8$^+$ T-cells are stimulated for

production due to tumor cells being eliminated by NK cells, and λ_{reg} serves as the inhibition term affecting NK cells and CD8$^+$ T-cells as influenced by Treg cells. Under conditions of hypoxia, the minimum activity levels for NK and CD8$^+$ T-cells, were employed, which subsequently increased linearly to the maximum observed levels under normoxic conditions (de Pillis, Radunskaya, and Wiseman 2005). The values of f_{NK} and m_{T8} were adjusted to reflect variations in oxygen levels. In accordance with experimental data (Barsoum et al. 2014), a 40-fold reduction in oxygen concentration (from 20% to 0.5%) resulted in a twofold increase in the apoptotic rate of immune cells. Furthermore, s_{CD4} serves as the source of CD4$^+$ T-cells, $\mu_{C_{d4}}$ represents the natural mortality rate of CD4$^+$ T-cells, re_{Cd4} indicates the growth rate of CD4$^+$ T-cells, and $C_{d4,max}$ signifies the maximum population of CD4$^+$ T-cells (Perelson, Kirschner, and De Boer 1993; Culshaw and Ruan 2000). The stimulation rate of CD8$^+$ T-cells by CD4$^+$ T-cells, as previously noted (De Palma and Jain 2017; Tian et al. 2017; de Pillis 2013), is represented by r_{Cd4}. The source term for CD4$^+$ T-cells, s_{CD4}, is reliant on the concentration of oxygen, as previous studies have indicated an eightfold decrease under hypoxic conditions (Wang et al. 2010). Moreover, a reduction in M2 TAMs led to an increase in the populations of CD8$^+$ T-cells and NK cells, while CD4$^+$ T-cells remained unaffected, according to experimental findings (Rolny et al. 2011), and these dynamics are articulated by the parameter λ_{M2}. The equations that describe the cell populations are normalized by transforming them dimensionless through the division of the cell count per finite element node by the initial quantity of cancer cells, T0=5×10² cells. The initial cancer cell population was established as follows: 98% CCs, 1% SCCs, and 1% ICCs (Hermann et al. 2007).

Additionally, the variable D represents the fractional cell kill of tumor cells by CD8$^+$ T-cells and is defined by Equation (S16) (de Pillis, Radunskaya, and Wiseman 2005; Milberg et al. 2019):

$$D = d_{im} \frac{\left(\frac{L}{T_{CCs}}\right)^{\lambda_{im}}}{s + \left(\frac{L}{T_{CCs}}\right)^{\lambda_{im}}} T_{CCs}, \tag{S16}$$

In this expression, d_{im} signifies the saturation threshold of fractional tumor cell eradication by CD8$^+$ T-cells, s reflects the steepness coefficient associated with the competition between the tumor and CD8$^+$ T-cells, and λ_{im} denotes the exponent corresponding to fractional tumor cell kill by CD8$^+$ T-cells.

Tumor Associated Macrophages (TAMs)

In this mathematical model, two distinct classifications of TAMs are recognized, specifically M1 and M2:

$$\frac{\partial M_1}{\partial t} = g_{m1}M_1 - m_{m1}M_1$$
$$\frac{\partial M_2}{\partial t} = g_{m2}M_2 - m_{m2}M_2 + r_{Cvegf,M2}C_{vegf}M_2 \quad (S17)$$

The parameters g_{m1} and g_{m2} signify the production rates for M1and M2 TAMs, which are influenced by oxygen concentrations, as indicated by prior investigations (Huang, Snuderl, and Jain 2011; Huang et al. 2013; Rolny et al. 2011) showing that a decline in hypoxia leads to a reorientation in TAM polarization from the M2-like to the M1-like phenotype. Previous investigations have established a correlation between TAMs and the expression of VEGF (Linde et al. 2012; Rolny et al. 2011; Stockmann et al. 2008). In particular, the overexpression of VEGF-A has been linked to an increased presence of M2-like TAMs ($r_{Cvegf,M2}$).

Implementation of Biphasic Theory for Understanding the Mechanical Behavior of the Tumor

The conservation principles of both the solid and fluid phases within the tumor system are expressed through the subsequent mass balance equations: (Roose et al. 2003; Voutouri and Stylianopoulos 2014)

$$\frac{\partial \Phi^c}{\partial t} + \nabla \cdot (v^s \Phi^c) = \frac{T_{CCs}}{T_{tot}} S_{CCs} + \frac{T_{SCCs}}{T_{tot}} S_{SCCs} + \frac{T_{ICCs}}{T_{tot}} S_{ICCs} \quad (S18)$$

$$\frac{\partial \Phi^f}{\partial t} + \nabla \cdot (v^f \Phi^f) = Q \quad (S19)$$

In this context, Φ^c and Φ^f represent the volume fractions of the solid and fluid phases, respectively, while v^s and v^f denote their associated velocities.

The variable Q in Equation (S20) signifies the fluid flux entering the tumor from blood vessels as well as from the adjacent normal tissue, subtracted by the fluid flux exiting via lymphatic vessels, and is expressed as (Stylianopoulos et al. 2013):

$$Q = L_p S_v (p_v - p_i) - L_{pl} S_{vl} (p_i - p_l) \quad (S20)$$

where L_p, S_v, and p_v are indicative of the hydraulic conductivity, vascular density, and vascular pressure, respectively; L_{pl}, S_{vl}, and p_l are the analogous parameters for lymphatic vessels; and p_i corresponds to the interstitial fluid pressure. In this particular model configuration and taking into account the principle of mass conservation within the tissue, the total of the solid and fluid volume fractions is established to consistently amount to one, in accordance with Equation (S21).

$$\Phi^f = 1 - \Phi^c \quad (S21)$$

Moreover, summing Equations (S18) and (S19), the mass balance can be expressed as follows:

$$\nabla \cdot \left(\Phi^c v^s + \Phi^f v^f \right) = Q + \frac{T_{CCs}}{T_{tot}} S_{CCs} + \frac{T_{SCCs}}{T_{tot}} S_{SCCs} + \frac{T_{ICCs}}{T_{tot}} S_{ICCs} \tag{S22}$$

where the fluid velocity v^f is defined according to Darcy's law (Byrne and Preziosi 2003)

$$\Phi^f (v^f - v^s) = -k_{th} \nabla p_i Þ v^f = \frac{-k_{th} \nabla p_i}{\Phi^f} + v^s \tag{S23}$$

with k_{th} denoting the hydraulic conductivity of the interstitial medium (Stylianopoulos et al. 2008).

In accordance with the biphasic theory applicable to soft tissues (Mow et al. 1980), the overall stress tensor $\boldsymbol{\sigma}_{tot}$ is constituted by the fluid phase stress tensor $\boldsymbol{\sigma}^f = -p_i \mathbf{I}$ and the solid phase stress tensor $\boldsymbol{\sigma}^s$. Consequently, the stress balance can be expressed as:

$$\nabla \cdot \boldsymbol{\sigma}_{tot} = \mathbf{0} \Rightarrow \nabla \cdot (\boldsymbol{\sigma}^s - p_i \mathbf{I}) = \mathbf{0} \tag{S24}$$

where the Cauchy stress tensor associated with the solid phase $\boldsymbol{\sigma}^s$ is delineated by: (Taber 2008)

$$\boldsymbol{\sigma}^s = J_e^{-1} \mathbf{F}_e \frac{\partial W}{\partial \mathbf{F}_e^T}, \tag{S25}$$

The mechanical properties of the tumor have been defined as incompressible and neo-Hookean, with the strain energy density represented by: (Ciarletta 2013; Voutouri et al. 2014; Xu, Bayly, and Taber 2009; Xu et al. 2010)

$$W = \frac{1}{2} \mu \left(-3 + II_1 \right) - p \left(J_e - 1_1 \right) - \left(\frac{p^2}{2k} \right) \tag{S26}$$

where μ and k denote the shear and bulk modulus of the material, respectively; J_e signifies the determinant of the elastic deformation gradient tensor $\mathbf{F}_e$; $II_1 = I_1 J_e^{-2/3}$ with $I_1 = \mathrm{tr}\mathbf{C}_e$ being the first invariant of the elastic Cauchy-Green deformation tensor $\mathbf{C}_e = \mathbf{F}_e^T \mathbf{F}_e$, and p is a penalty variable introduced for materials that exhibit near incompressibility that regularizes the constraint involved in the second term (Holzapfel, Gasser, and Ogden 2000). The adjacent normal tissue is presumed to be compressible and neo-Hookean, characterized by a Poisson ratio of 0.2.

Functional Vascular Density

To assess the functional vascular density, it is claimed that is impacted by the decrease in the diameter of blood vessels ($\frac{d}{d_0}$) linked to the increase of solid stress (Mpekris et al. 2015). Furthermore, the functional vascular density is dependent on the permeability of the tumor vascular wall (Stylianopoulos and Jain 2013), as hyper-permeable vessels diminish both perfusion and overall functionality of the vessels.

The functional vascular density can be expressed as:

$$S_v = \frac{d}{d_0} S_v^0 \rho_v^{EC}, \tag{S27}$$

where S_v^0 will be determined by the dimensions of the pores in the vascular wall (i.e., its permeability) and ρ_v^{EC} is related to the density of endothelial cells.

Transport of Oxygen

The variation in oxygen levels within the tumor tissue is considered to be based on both its transport mechanisms, namely convection and diffusion, alongside the rate of oxygen utilized by the cells, as well as the influx of oxygen from the surrounding blood vessels (Kim, Stolarska, and Othmer 2011; Roose et al. 2003), specifically described by the equation:

$$\frac{\partial c_{ox}}{\partial t} + \nabla \cdot \left(c_{ox} v^f \right) = D_{ox} \nabla^2 c_{ox} - \frac{A_{ox} c_{ox}}{c_{ox} + k_{ox}} T_{tot} + P_{er} S_V \left(C_{iox} - c_{ox} \right), \tag{S28}$$

where c_{ox} represents the oxygen concentration, D_{ox} denotes the diffusion coefficient for oxygen in the interstitial region, A_{ox} and k_{ox} are parameters associated with oxygen uptake, P_{er} indicates the vascular permeability of oxygen, which governs the diffusion across the walls of tumor vessels, and C_{iox} is signifies the oxygen concentration present in the vascular system. The transport of oxygen across the vascular barrier is primarily governed by diffusion, as the contribution of convection is minimal in comparison to diffusion (Popel 1989). In view of the uniform reduction of interstitial fluid pressure observed in tumors, the pressure differentials both within the tissue and across the tumor vessel wall are minimal (Chauhan et al. 2011), leading to an expectation of low Péclet numbers.

Components of Tumor Vasculature

In accordance with our mathematical model, we integrate the constituents of tumor vasculature, which include the endothelial cells, the vascular endothelial growth factor (VEGF), as well as Angiopoietin 1 and Angiopoietin 2.

Transport Equation of Endothelial Cells

The flux of endothelial cells is represented by the Equation (S29): (Schugart et al. 2008)

$$\frac{\partial \hat{e}}{\partial t} = \nabla \cdot (D_{EC}(\alpha 1, \alpha 2)\nabla \hat{e} - x_n \hat{e} H(1-\hat{e}) C^0_{vegf} \nabla \hat{C}_{vegf} + \frac{1}{e_0}(\lambda_2 C^0_{vegf} e_0 \hat{C}_{vegf} \hat{e}) H(1-\hat{e}) - (\lambda_4 e_0 \hat{e})\hat{e} \quad \text{(S29)}$$

The proliferation of endothelial cells is influenced by the concentration of VEGF, as well as the density of endothelial cells. $\hat{e}$ is the dimensionless density of endothelial cells. $\hat{C}_{vegf}$ and C^0_{vegf} are dimensionless and refer to VEGF concentrations. The diffusion coefficient of endothelial cells is contingent upon Ang1 and Ang2, described by $D_{EC}(\alpha 1, \alpha 2) = D_{EC}(1+s1\alpha 1)^{-\alpha}(1+s2\alpha 2)^{b}$, where α and b are set to unity (Plank, Sleeman, and Jones 2004), x_n denotes a chemotactic factor. The dimensionless concentration of endothelial cells is derived by normalizing with the reference concentration, $\hat{e} = \frac{e}{e_0}$. The constants λ_2 and λ_4 are positive parameters.

Transport Equation of VEGF

The concentration of Vascular Endothelial Growth Factor (VEGF) is influenced by various factors including diffusion, synthesis from cancer cells in hypoxic conditions, and interaction with endothelial cell receptors (Schugart et al. 2008). The concentration of VEGF is represented by the following Equation (S30):

$$\frac{\partial \hat{e}}{\partial t} = \nabla \cdot (D_{EC}(\alpha 1, \alpha 2)\nabla \hat{e} - x_n \hat{e} H(1-\hat{e}) C^0_{vegf} \nabla \hat{C}_{vegf} + \frac{1}{e_0}(\lambda_2 C^0_{vegf} e_0 \hat{C}_{vegf} \hat{e}) H(1-\hat{e}) - (\lambda_4 e_0 \hat{e})\hat{e} \quad \text{(S30)}$$

where $\hat{C}_{vegf}$ denotes the dimensionless VEGF concentration achieved by normalizing with a reference value $\hat{C}_{vegf} = \frac{C_{vegf}}{C^0_{vegf}}$ and $\hat{c}_{ox}$ represents the dimensionless oxygen concentration normalized as: $\hat{c}_{ox} = \frac{c_{ox}}{c^0_{ox}}$.

It is assumed that VEGF is exclusively synthesized by tumor cells, with its production being intensified in response to hypoxic conditions, as characterized by the oxygen tension parameter G_α (Schugart et al. 2008).

$$G_\alpha(\hat{c}_{ox}) = \begin{Bmatrix} 3\hat{c}_{ox}, \text{for } 0 < \hat{c}_{ox} < 0.5 \text{ (hypoxia)} \\ 2 - \hat{c}_{ox}, \text{for } 0.5 < \hat{c}_{ox} < 1 \text{ (normoxia)} \\ \hat{c}_{ox}, \text{for } 1 < \hat{c}_{ox} \text{ (hyperoxia)} \end{Bmatrix} \quad \text{(S31)}$$

VEGF becomes unavailable through its binding to receptors on endothelial cells, and it is also capable of diffusing within the tumor characterized by a diffusion coefficient D_{VEGF}. λ_{10}, λ_{11} and λ_{13} are defined as positive constants. Furthermore, the removal of CD4^{+} T cells led to a notable upregulation of VEGF ($\lambda_{CD4,Cvegf} C_{d4} \widehat{C}_{vegf}$) without marked alterations in Ang1-Ang2 levels (Tian et al. 2017).

Transport Equation of Angiopoietin 1(Ang1) and Angiopoietin 2 (Ang2)

The synthesis of the Angiopoietin 1 (Ang1) and Angiopoietin 2 (Ang2) is augmented under hypoxic conditions, which correlate with levels of vascular endothelial growth factor (VEGF) (Plank, Sleeman, and Jones 2004). Both angiopoietin 1 (Ang1, α_1) and angiopoietin 2 (Ang2, α_2) exhibit up-regulation in response to hypoxia and are secreted by endothelial cells.

$$\frac{\partial \alpha_1}{\partial t} = \frac{b_1}{\alpha_1^0} p_C + \mu_1 (1 - \alpha_1) \tag{S32}$$

$$\frac{\partial \hat{\alpha}_2}{\partial t} = \frac{b_2}{\alpha_2^0} G_\alpha (\hat{c}_{ox}) \hat{e} e_0 - \mu_2 \hat{\alpha}_2 \tag{S33}$$

In this context, b_1, b_2, μ_1, and μ_2 are defined as positive constants. The dimensionless forms of Ang1 and Ang2 are derived through normalization with respect to a reference concentration, $\hat{\alpha}_1 = \frac{\alpha_1}{\alpha_1^0}$ and $\hat{\alpha}_2 = \frac{\alpha_2}{\alpha_2^0}$. The term for oxygen tension, denoted as G_α, corresponds to the parameters utilized for VEGF. To simplify the equations, we omit considerations of the diffusion processes associated with Ang1 and Ang2, as well as their interactions with specific Tie receptors (Billy et al. 2009; Gevertz and Torquato 2006).

Transport of Drugs

Calculation of hydraulic conductivity, vascular permeability, reflection coefficient and surviving fractions of tumor cells

The hydraulic conductivity was determined from the specified relation (Deen 1987):

$$L_p = \frac{\gamma r_0^2}{8 \eta L_{vw}} \tag{S34}$$

where γ signifies the fraction of vessel wall surface area that is occupied by pores, r_0 represents the pore radius, η refers to the viscosity of water at 310K, and L_{vw} indicates the thickness of the vessel wall.

The vascular permeability P_{er} alongside the reflection coefficient σ_f were ascertained from the following equations:

$$P_{er} = \frac{\gamma H D_0}{L_{vw}} \quad \text{(S35)}$$

$$\sigma_f = 1 - W \quad \text{(S36)}$$

respectively, where H and W account for hydrodynamic as well as electrostatic interactions, and D_0 is recognized as the diffusion coefficient of a particle in an free solution at 310K, as delineated by the Stokes-Einstein relationship:

$$D_0 = \frac{K_b T_{emp}}{6\pi\eta r_s} \quad \text{(S37)}$$

where K_b represents the Boltzmann constant, T_{emp} denotes temperature, and r_s refers to the radius of the diffusing particle. By disregarding electrostatic interactions, H and W are simplified to (Deen 1987):

$$H = \frac{6\pi F}{K_t} \quad \text{(S38)}$$

$$W = \frac{F(2-F)K_s}{2K_t} \quad \text{(S39)}$$

where F is defined as the partition coefficient (Deen 1987):

$$F = 1 - \lambda^2 \quad \text{(S40)}$$

and λ constitutes the ratio of the drug size to the vessel wall pore size. The coefficients K_s and K_t as presented in Equations (S38) and (S39) are determined by:

$$\begin{pmatrix} K_t \\ K_s \end{pmatrix} = \frac{9}{4}\pi^2\sqrt{2}(1-\lambda)^{-5/2}\left[1 + \sum_{n=1}^{2}\begin{pmatrix} \alpha_n \\ b_n \end{pmatrix}(1-\lambda)^n\right] + \sum_{n=0}^{4}\begin{pmatrix} \alpha_{n+3} \\ b_{n+3} \end{pmatrix}\lambda^n \quad \text{(S41)}$$

In relation to oxygen, γ and H in Equation (S35) were established as equal to unity, thereby presuming that oxygen can diffuse through any point of the vessel wall without being impeded by hydrodynamic interactions; D_0 for oxygen was assumed to be equivalent to the diffusion coefficient within the tissue, specifically:

$$P_{erox} = \frac{D_0}{L_{vw}} \tag{S42}$$

To clarify the impact of drug delivery on tumor growth, the parameter representing the surviving fraction of cells, denoted as S_f, is incorporated into Equation (S5), such that in the scenario devoid of pharmacological agents, S_f is equivalent to unity. The fraction of surviving cells in relation to the concentration of the drug has been previously assessed through empirical methods for doxorubicin (Kerr et al. 1986), and the resultant data were subsequently fitted to an exponential model as a function of the internalized chemotherapeutic concentration, c_{int}, namely,

$$S_{urf} = \exp(-\omega c_{int}) \tag{S43}$$

wherein represents a fitting parameter for doxorubicin (Eikenberry 2009). In accordance with this mathematical formulation, should we disregard immune cells and suggest that CCs, SCCs, and ICCs are eliminated by chemotherapy, the terms S_{CCs}, S_{SCCs} and S_{ICCs} in Equations (S9), (S10) and (S11) would converge to zero, subsequently rendering the growth stretch ratio in Equation (S4) a constant value. It is reasonable to expect that the growth stretch ratio would decrease following the cessation of CCs, SCCs, and ICCs, rather than sustaining a stable value. To account for this, Equation (S43) was modified in the form:

$$S_f = 2*(\exp(\omega c_{int}) - 0.5) \tag{S44}$$

Given that cancer stem cells (SCCs) exhibit chemoresistance, the equation is calibrated to align with empirical observations (Liu et al. 2006) in order to determine the fitting parameter ω pertinent to this context (S_{fSCCs}).

2 Supplementary Figures and Tables

2.1 Supplementary Figures

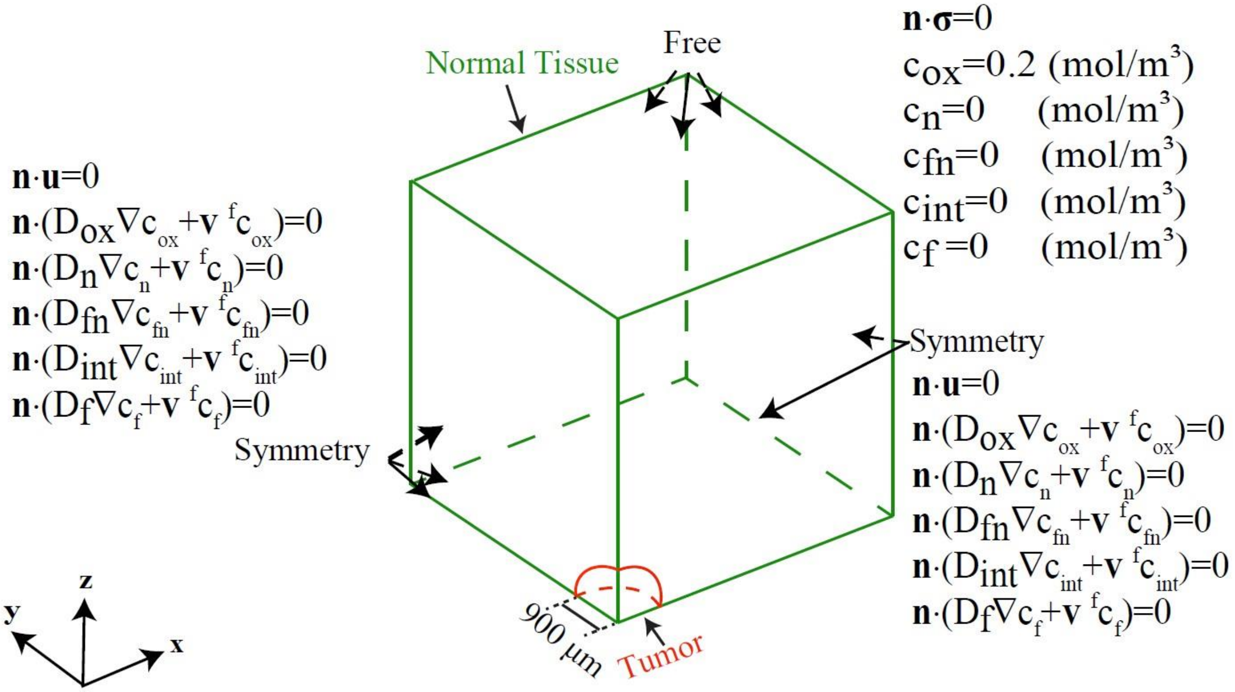

Supplementary Figure 1. The computational domain along with the specified boundary conditions that have been utilized for the current analysis of stress (**σ**), displacement (**u**), the concentration of oxygen (c_{ox}), and the concentrations associated with the nanotherapeutic agent Doxil, specifically denoted as c_n, c_{fn}, and c_{int}, in addition to the immunotherapeutic agent anti-PD-1, represented by c_f.

Model Assumptions

- Tumor tissue is presented as an incompressible neo-Hookean material.
- The tumor-host environment is modeled as a biphasic medium composed of a solid phase (including cells and extracellular matrix) and a fluid phase (interstitial fluid).
- The multiplicative decomposition of the deformation gradient tensor is used to represent tumor growth.
- The model is developed within a continuum mechanics framework and computed using the finite elements method (FEM).

↓

Input Parameters

- Mechanotherapy-related changes, including variations in tumor shear and bulk modulus, as well as alterations in hydraulic conductivity.
- Sonopermeation-induced effects, such as modifications in vessel wall pore size (Equations 6–7), changes in functional vascular density within the tumor (Equation S27), and shear stresses exerted on endothelial cells by microbubbles (Equations 8–10).
- Drug-specific properties, including diffusion coefficients and pharmacokinetic characteristics.

↓

Governing Equations

- **Growth kinematics:** tumor expansion modeled via multiplicative decomposition of the deformation gradient (Equations S1-S4).
- **Momentum balance:** biphasic theory for solid and fluid phases (Eq. S18–S26).
- **Mass balance of diluted species:** nano-immunotherapy (Equations 1-5) & (Equations S34-S44), oxygenation (Equation S28)).

↓

Numerical Methods for Solving the Mathematical Model

- Multiple simulations conducted using COMSOL Multiphysics.
- The mathematical model is solved using the FEM for spatial discretization
- Time integration is performed using the Backward Differentiation Formula (BDF) method.

↓

Model Output Variables

- **Tissue deformation** resulting from tumor growth.
- **Tumor perfusion**, including functional vascular density & oxygen levels.
- **Hypoxia distribution in tumor tissue.**

Supplementary Figure 2. This diagram presents the computational framework designed to assess therapeutic outcomes resulting from mechanotherapy and sonopermeation in solid tumors. It summarizes the model's core assumptions and the primary input parameters used in the mathematical formulation. The flowchart includes the governing equations—with corresponding equation numbers—and the numerical methods applied to solve them. Finally, it highlights the key output variables used to evaluate treatment efficacy.

2.2 Supplementary Tables

Supplementary Table 1. Parameter values applied within the model.

Description	Parameter	Value [Units]	Reference
Hydraulic conductivity	k_{th}	6.5×10^{-11} [$m^2\cdot Pa^{-1}\cdot day^{-1}$] for the host tissue,<br>$6.5\times10^{-11}$ [$m^2\cdot Pa^{-1}\cdot day^{-1}$] for the tumor,<br>$6.5\times10^{-9}$ [$m^2\cdot Pa^{-1}\cdot day^{-1}$] for the tumor with the effect of ketotifen	(Netti et al. 2000; Papageorgis et al. 2017)
Shear modulus	μ	21 [kPa] for the host tissue,<br>60 [kPa] for the tumor,<br>30 [kPa] for the tumor with the effect of ketotifen	(Eder et al. 2014; Netti et al. 2000; Samani, Zubovits, and Plewes 2007; Mpekris et al. 2024; Panagi et al. 2024)
Bulk modulus	k	28 [kPa] for the host tissue,<br>580 [kPa] for the tumor,<br>290 [kPa] for the tumor with the effect of ketotifen	(Eder et al. 2014; Netti et al. 2000; Samani, Zubovits, and Plewes 2007; Mpekris et al. 2024; Panagi et al. 2024)
Internalization rate of the drug by the cells	k_{int}	3.7 [day^{-1}]	(Mok et al. 2009; Schmidt and Wittrup 2009)
Rate constant for the chemotherapy release	k_{el}	0.181[day^{-1}]	----
Degradation rate of the chemotherapeutic agent	k_{deg}	0.02[1/h]	(Wu and Ofner 2013)
Degradation rate constant of the free antibody	k_{deg_i}	0.002[1/h]	adaptation to the model's data

Chemotherapy molecules contained in the nanocarrier	α	10^4	(Dawidczyk et al. 2014)
Radius of the diffusing nanoparticle particle	r_s	$50x10^{-9}$ [m]	(Pluen et al. 2001)
Radius of the antibody (anti-PD-L1) used in immunotherapy	r_{s_i}	6.35 $x10^{-9}$ [m]	(Tykodi et al. 2012)
Drug diffusion coefficient	D_f	8.64×10^{-6} [$m^2\cdot day^{-1}$]	(Pluen et al. 2001)
Nanoparticle diffusion coefficient	D_n	8.64×10^{-6} [$m^2 \cdot day^{-1}$] for 1[nm] drug;<br>8.64×10^{-8} [$m^2 \cdot day^{-1}$] for 50 [nm] drug	(Pluen et al. 2001)
Cancer cell survival constant	ω	0.6603 [m^3/mol]	(Eikenberry 2009)
Stem-cell-like cell survival constant	ω_{SCCs}	0.0272 [m^3/mol]	(Eikenberry 2009; Liu et al. 2006)
Induced cancer cell survival constant	ω_{ICCs}	0.0272 [m^3/mol]	----
Blood circulation decay	k_d	0.417 [day^{-1}]	(Chauhan et al. 2012)
Vessel wall thickness	L_{vw}	5×10^{-6} [m]	(Stylianopoulos et al. 2013)
Water viscosity at 310K	η	7×10^{-4} [Pa·s]	(Stylianopoulos et al. 2013)
Absolute temperature	T_{emp}	310 [K]	----
Fraction of vessel wall surface area occupied by pores	γ	1×10^{-5} [-]	(Chauhan et al. 2012)
Mechanical Index of transducer	MI	0.34	(Mpekris et al. 2024; Neophytou et al. 2024)

Frequency used for sonopermeation	fr	2 [MHz]	(Snipstad et al. 2018; Lewin and Bjørnø 1982)
Density of the liquid medium (i.e. the blood) surrounding the pulsating shelled microbubble	ρ_L	1000 [kg/m^3]	(Cowley and McGinty 2019)
Viscosity of the liquid medium (i.e. the blood) surrounding the pulsating shelled microbubble	μ_L	10^{-3} [Pa·s]	(Cowley and McGinty 2019)
Displacement amplitude of the microbubble wall	η_m	0.2×10^{-6} [m]	(Lewin and Bjørnø 1982)
Equilibrium radius of the shelled microbubble	R_0	2 [μm]	(Lewin and Bjørnø 1982)
Fractional tumor cell killing by NK cells	c	range *: 3.23×10^{-7} -3.23×10^{-6} [cell^{-1}·day^{-1}]	(de Pillis, Radunskaya, and Wiseman 2005)
Cell diffusion coefficient	D_{cell}	1.5x10^{-11} [m^2/s]	(Tracqui 1995; Voutouri et al. 2019)
Stem-cancer cell growth multiplier	α_{SCCs}	range **: 1-2 [-]	(Burroughs et al. 2011)
Induced cancer cell growth multiplier	α_{ICCs}	range **: 1-2 [-]	---
Rate of transition from non-stem cancer cells to stem cancer cells	p_{TC}	0.55 [day^{-1}]	(Goldman et al. 2015)
Rate of transition from stem cancer cells to non-stem cancer cells	p_{CT}	1 [day^{-1}]	(Goldman et al. 2015)

Rate of transition from stem cancer cells to induced cancer cells	p_{CI}	0.58 [day^{-1}]	(Goldman et al. 2015)
Rate of transition from induced cancer cells to stem cancer cells	p_{IC}	0.96 [day^{-1}]	(Goldman et al. 2015)
Rate of transition from non-stem cancer cells to induced cancer cells	p_{TI}	0.21 [day^{-1}]	(Goldman et al. 2015)
Rate of transition from induced cancer cells to non-stem cancer cells	p_{IT}	1 [day^{-1}]	(Goldman et al. 2015)
Tumoricidal effect of M1 TAMs in cancer cells	λ_{M1}	3 [d^{-1}]	(Mahlbacher et al. 2018)
Growth rate parameter	k_1	3.50 [day^{-1}]	---
Growth rate parameter	k_2	0.0083 [$mol \cdot m^{-3}$]	(Casciari, Sotirchos, and Sutherland 1992b)
Constant source of NK cells	σ_{nk}	1.3×10^4 [$cell \cdot day^{-1}$]	(de Pillis, Radunskaya, and Wiseman 2005)
Death rate of NK cells	f_{NK}	range** 0.0412 - 0.0814 [day^{-1}]	(de Pillis, Radunskaya, and Wiseman 2005)
Recruitment rate of NK cells	g_{NK}	0.025 [day^{-1}]	(de Pillis, Radunskaya, and Wiseman 2005)
Steepness coefficient of NK cell recruitment curve	h	2.02×10^7 [$cell^2$]	(de Pillis, Radunskaya, and Wiseman 2005)
Inactivation rate of NK cells	p_{im}	1×10^{-7} [$cell^{-1} \cdot day^{-1}$]	(de Pillis, Radunskaya, and Wiseman 2005)

Inhibition term of NK cells and $CD8^+$ T-cells from Treg cells	λ_{reg}	100 [$cell^{-1}\cdot day^{-1}$]	(Fouchet and Regoes 2008)
Death rate of $CD8^+$ T-cells	m_{T8}	range **: 0.02 - 0.04 [day^{-1}]	(de Pillis, Radunskaya, and Wiseman 2005)
Death rate of regulatory T-cells	m_{reg}	0.02 [day^{-1}]	(Fouchet and Regoes 2008)
Recruitment rate of $CD8^+$ T-cells	j_{T8}	0.0375 [day^{-1}]	(de Pillis, Radunskaya, and Wiseman 2005)
Recruitment rate of regulatory T-cells	g_{reg}	0.0375 [day^{-1}]	(Fouchet and Regoes 2008)
Steepness coefficient of $CD8^+$ T-cells recruitment curve	k_{im}	2.02×10^{7} [$cell^{2}$]	(de Pillis, Radunskaya, and Wiseman 2005)
Inactivation rate of $CD8^+$ T-cells	q	3.42×10^{-10} [$cell^{-1}\cdot day^{-1}$]	(de Pillis, Radunskaya, and Wiseman 2005)
Stimulation rate of $CD8^+$ T-cells	r	1.1×10^{-7} [$cell^{-1}\cdot day^{-1}$]	(de Pillis, Radunskaya, and Wiseman 2005)
Source term of $CD4^+$ T-cells	s_{CD4}	150 [day^{-1}] **	(Perelson, Kirschner, and De Boer 1993)
Natural death rate of $CD4^+$ T-cells	$\mu_{C_{d4}}$	0.02 [day^{-1}]	(Perelson, Kirschner, and De Boer 1993)
Growth rate of $CD4^+$ T-cells	re_{Cd4}	0.03 [day^{-1}]	(Perelson, Kirschner, and De Boer 1993)
Stimulation rate of $CD8^+$ T cells by $CD4^+$ T-cells	r_{Cd4}	$1x10^{-15}$ [$cells^{-1}.day^{-1}$]	(de Pillis, Radunskaya, and Wiseman 2005)

Source term of CD8+ T-cells	σ_{T8}	150 [day^{-1}]	----
Fractional tumor cell killing by CD8+ T-cells	d_{im}	range * : 1.43 – 7.15 [day^{-1}]	(de Pillis, Radunskaya, and Wiseman 2005)
Exponent of fractional cell kill by CD8+ T-cells	λ_{im}	1.36 [-]	(de Pillis, Radunskaya, and Wiseman 2005)
Steepness coefficient of the tumor-CD8+ T-cells competition term	s	2.73 [-]	(de Pillis, Radunskaya, and Wiseman 2005)
Death rate of regulatory M1 TAMs	m_{m1}	0.02 [day^{-1}]	----
Death rate of regulatory M2 TAMs	m_{m2}	0.02 [day^{-1}]	----
Initial oxygen concentration	C_{iox}	0.2 [mol·m^{-3}]	(Mpekris et al. 2017)
Oxygen diffusion coefficient	D_{ox}	1.55×10^{-4} [m^2·day^{-1}]	(Kim, Stolarska, and Othmer 2011)
Oxygen uptake	A_{ox}	2200 [mol·m^{-3}·day^{-1}]	(Casciari, Sotirchos, and Sutherland 1992b; Kim, Stolarska, and Othmer 2011)
Oxygen uptake	k_{ox}	0.00464 [mol·m^{-3}]	(Casciari, Sotirchos, and Sutherland 1992b; Kim, Stolarska, and Othmer 2011)
Endothelial cell diffusion coefficient	D_{EC}	1x10^{-15} [m^2/s]	(Plank, Sleeman, and Jones 2004)

Chemotactic endothelial cell	x_n	$2x10^{-15}$ [m^5/kg-s]	(Schugart et al. 2008)
Reference value of endothelial cell	e_0	$1x10^{-3}$ [g/cm^3]	(Schugart et al. 2008)
Positive parameter	λ_2	$1x10^{-5}$ [cm^3/g-s]	(Schugart et al. 2008)
Positive parameter	λ_4	$1x10^{-1}$ [cm^3/g-s]	(Schugart et al. 2008)
Positive parameter	s1	$1x10^3$ [cm^3/ g]	(Plank, Sleeman, and Jones 2004)
Positive parameter	s2	$1x10^3$ [cm^3/g]	(Plank, Sleeman, and Jones 2004)
VEGF diffusion coefficient	D_{VEGF}	$3.1x10^{-11}$ [m^2/s]	(Schugart et al. 2008)
Reference VEGF concentration	C^0_{vegf}	$1x10^{-3}$ [g/cm^3]	(Schugart et al. 2008)
Positive parameter	λ_{10}	$6.8x10^{-3}$ [1/s]	(Schugart et al. 2008)
Positive parameter	λ_{11}	4 [cm^3/g-s]	(Schugart et al. 2008)
Positive parameter	λ_{13}	$4x10^{-5}$ [1/s]	(Schugart et al. 2008)
Reference α_1 concentration	α_1^0	$1x10^{-3}$ [g/cm^3]	(Plank, Sleeman, and Jones 2004)
Reference α_2 concentration	α_2^0	$1x10^{-3}$ [g/cm^3]	(Plank, Sleeman, and Jones 2004)
Positive parameter	b_1	2280 [1/h]	(Plank, Sleeman, and Jones 2004)
Positive parameter	b_2	18240 [1/h]	(Plank, Sleeman, and Jones 2004)

Positive parameter	μ_1	456 [1/h]	(Plank, Sleeman, and Jones 2004)
Positive parameter	μ_2	456 [1/h]	(Plank, Sleeman, and Jones 2004)

* : linear increase from minimum to maximum value depending on oxygen levels

** : linear decrease from maximum to minimum value depending on oxygen levels

Supplementary Table 2. The value of the parameter k_1, which is employed in the process of fitting the mathematical model to the experimental data for each cancer cell line.

Experimental study	k_1
4T1-breast tumor cells (Neophytou et al. 2024)	0.54 day^{-1}
E0771-breast tumor cells (Neophytou et al. 2024)	0.56 day^{-1}

References

Ambrosi, D., and F. Mollica. 2002. 'On the mechanics of a growing tumor', *International Journal of Engineering Science*, 40: 1297-316.

Barsoum, I. B., C. A. Smallwood, D. R. Siemens, and C. H. Graham. 2014. 'A mechanism of hypoxia-mediated escape from adaptive immunity in cancer cells', *Cancer Res*, 74: 665-74.

Billy, F., B. Ribba, O. Saut, H. Morre-Trouilhet, T. Colin, D. Bresch, J. P. Boissel, E. Grenier, and J. P. Flandrois. 2009. 'A pharmacologically based multiscale mathematical model of angiogenesis and its use in investigating the efficacy of a new cancer treatment strategy', *J Theor Biol*, 260: 545-62.

Burroughs, N. J., B. M. P. M. Oliveira, A. A. Pinto, and M. Ferreira. 2011. 'Immune response dynamics', *Mathematical and Computer Modelling*, 53: 1410-19.

Byrne, H., and L. Preziosi. 2003. 'Modelling solid tumour growth using the theory of mixtures', *Mathematical medicine and biology : a journal of the IMA*, 20: 341-66.

Casciari, J. J., S. V. Sotirchos, and R. M. Sutherland. 1992a. 'Mathematical modelling of microenvironment and growth in EMT6/Ro multicellular tumour spheroids', *Cell Prolif*, 25: 1-22.

———. 1992b. 'Variations in tumor cell growth rates and metabolism with oxygen concentration, glucose concentration, and extracellular pH', *J Cell Physiol*, 151: 386-94.

Chauhan, V. P., T. Stylianopoulos, Y. Boucher, and R. K. Jain. 2011. 'Delivery of molecular and nanomedicine to tumors: Transport barriers and strategies', *Annual Reviews Chemical and Biomolecular Engineering*, 2: 281-98.

Chauhan, V. P., T. Stylianopoulos, J. D. Martin, Z. Popovic, O. Chen, W. S. Kamoun, M. G. Bawendi, D. Fukumura, and R. K. Jain. 2012. 'Normalization of tumour blood vessels improves the delivery of nanomedicines in a size-dependent manner', *Nature Nanotechnology*, 7: 383-88.

Ciarletta, P. 2013. 'Buckling instability in growing tumor spheroids', *Phys Rev Lett*, 110: 158102.

Conley, S. J., E. Gheordunescu, P. Kakarala, B. Newman, H. Korkaya, A. N. Heath, S. G. Clouthier, and M. S. Wicha. 2012. 'Antiangiogenic agents increase breast cancer stem cells via the generation of tumor hypoxia', *Proc Natl Acad Sci U S A*, 109: 2784-9.

Cowley, J., and S. McGinty. 2019. 'A mathematical model of sonoporation using a liquid-crystalline shelled microbubble', *Ultrasonics*, 96: 214-19.

Culshaw, Rebecca V, and Shigui Ruan. 2000. 'A delay-differential equation model of HIV infection of CD4+ T-cells', *Mathematical biosciences*, 165: 27-39.

Dawidczyk, C. M., C. Kim, J. H. Park, L. M. Russell, K. H. Lee, M. G. Pomper, and P. C. Searson. 2014. 'State-of-the-art in design rules for drug delivery platforms: lessons learned from FDA-approved nanomedicines', *J Control Release*, 187: 133-44.

De Palma, M., and R. K. Jain. 2017. 'CD4(+) T Cell Activation and Vascular Normalization: Two Sides of the Same Coin?', *Immunity*, 46: 773-75.

de Pillis, L. G., A. E. Radunskaya, and C. L. Wiseman. 2005. 'A validated mathematical model of cell-mediated immune response to tumor growth', *Cancer Research*, 65: 7950-58.

de Pillis, Lisette G. 2013. 'Mathematical modeling of the regulatory T cell effects on renal cell carcinoma treatment'.

Deen, W. M. 1987. 'Hindered Transport of Large molecules in Liquid-Filled Pores', *AIChE J*, 33: 1409-25.

Eder, M., S. Raith, J. Jalali, A. Volf, M. Settles, H. G. Machens, and L. Kovacs. 2014. 'Comparison of different material models to simulate 3-d breast deformations using finite element analysis', *Ann Biomed Eng*, 42: 843-57.

Eikenberry, S. 2009. 'A tumor cord model for doxorubicin delivery and dose optimization in solid tumors', *Theor Biol Med Model*, 6: 16.

Fouchet, D., and R. Regoes. 2008. 'A population dynamics analysis of the interaction between adaptive regulatory T cells and antigen presenting cells', *PLoS One*, 3: e2306.

Gevertz, J. L., and S. Torquato. 2006. 'Modeling the effects of vasculature evolution on early brain tumor growth', *J Theor Biol*, 243: 517-31.

Goldman, A., B. Majumder, A. Dhawan, S. Ravi, D. Goldman, M. Kohandel, P. K. Majumder, and S. Sengupta. 2015. 'Temporally sequenced anticancer drugs overcome adaptive resistance by targeting a vulnerable chemotherapy-induced phenotypic transition', *Nat Commun*, 6: 6139.

Hermann, P. C., S. L. Huber, T. Herrler, A. Aicher, J. W. Ellwart, M. Guba, C. J. Bruns, and C. Heeschen. 2007. 'Distinct populations of cancer stem cells determine tumor growth and metastatic activity in human pancreatic cancer', *Cell Stem Cell*, 1: 313-23.

Holzapfel, G. A., T. C. Gasser, and R. W. Ogden. 2000. 'A new constitutive framework for arterial wall mechanics and a comparative study of material models', *J. Elasticity*, 61: 1-48.

Huang, Y., M. Snuderl, and R. K. Jain. 2011. 'Polarization of tumor-associated macrophages: a novel strategy for vascular normalization and antitumor immunity', *Cancer Cell*, 19: 1-2.

Huang, Y., T. Stylianopoulos, D. G. Duda, D. Fukumura, and R. K. Jain. 2013. 'Benefits of vascular normalization are dose and time dependent--letter', *Cancer Res*, 73: 7144-6.

Kerr, D. J., A. M. Kerr, R. I. Freshney, and S. B. Kaye. 1986. 'Comparative intracellular uptake of adriamycin and 4'-deoxydoxorubicin by non-small cell lung tumor cells in culture and its relationship to cell survival', *Biochem Pharmacol*, 35: 2817-23.

Kim, Y., M. A. Stolarska, and H. G. Othmer. 2011. 'The role of the microenvironment in tumor growth and invasion', *Progress in biophysics and molecular biology*, 106: 353-79.

Lewin, P.A., and L. Bjørnø. 1982. 'Acoustically induced shear stresses in the vicinity of microbubbles in tissue', *J. Acoust. Soc. Am.*, 71: 728-34.

Linde, N., W. Lederle, S. Depner, N. van Rooijen, C. M. Gutschalk, and M. M. Mueller. 2012. 'Vascular endothelial growth factor-induced skin carcinogenesis depends on recruitment and alternative activation of macrophages', *J Pathol*, 227: 17-28.

Liu, G., X. Yuan, Z. Zeng, P. Tunici, H. Ng, I. R. Abdulkadir, L. Lu, D. Irvin, K. L. Black, and J. S. Yu. 2006. 'Analysis of gene expression and chemoresistance of CD133+ cancer stem cells in glioblastoma', *Mol Cancer*, 5: 67.

MacLaurin, J., J. Chapman, G. W. Jones, and T. Roose. 2012. 'The buckling of capillaries in solid tumours', *Proc. R. Soc. A*, 468: 4123-45.

Mahlbacher, G., L. T. Curtis, J. Lowengrub, and H. B. Frieboes. 2018. 'Mathematical modeling of tumor-associated macrophage interactions with the cancer microenvironment', *J Immunother Cancer*, 6: 10.

Mascheroni, Pietro, Melania Carfagna, Alfio Grillo, DP Boso, and Bernhard A Schrefler. 2018. 'An avascular tumor growth model based on porous media mechanics and evolving natural states', *Mathematics and Mechanics of Solids*, 23: 686-712.

Milberg, O., C. Gong, M. Jafarnejad, I. H. Bartelink, B. Wang, P. Vicini, R. Narwal, L. Roskos, and A. S. Popel. 2019. 'A QSP Model for Predicting Clinical Responses to Monotherapy, Combination and Sequential Therapy Following CTLA-4, PD-1, and PD-L1 Checkpoint Blockade', *Sci Rep*, 9: 11286.

Mok, W., T. Stylianopoulos, Y. Boucher, and R. K. Jain. 2009. 'Mathematical modeling of herpes simplex virus distribution in solid tumors: implications for cancer gene therapy', *Clin Cancer Res*, 15: 2352-60.

Mow, V. C., S. C. Kuei, W. M. Lai, and C. G. Armstrong. 1980. 'Biphasic creep and stress relaxation of articular cartilage in compression? Theory and experiments', *Journal of Biomechanical Engineering*, 102: 73-84.

Mpekris, F., S. Angeli, A. P. Pirentis, and T. Stylianopoulos. 2015. 'Stress-mediated progression of solid tumors: effect of mechanical stress on tissue oxygenation, cancer cell proliferation, and drug delivery', *Biomech Model Mechanobiol*, 14: 1391-402.

Mpekris, F., J. W. Baish, T. Stylianopoulos, and R. K. Jain. 2017. 'Role of vascular normalization in benefit from metronomic chemotherapy', *Proc Natl Acad Sci U S A*, 114: 1994-99.

Mpekris, F., M. Panagi, A. Charalambous, C. Voutouri, C. Michael, A. Papoui, and T. Stylianopoulos. 2024. 'A synergistic approach for modulating the tumor microenvironment to enhance nano-immunotherapy in sarcomas', *Neoplasia*, 51: 100990.

Mpekris, F., C. Voutouri, P. Papageorgis, and T. Stylianopoulos. 2018. 'Stress alleviation strategy in cancer treatment: Insights from a mathematical model', *Z Angew Math Mech*: 1-12.

Neophytou, C., A. Charalambous, C. Voutouri, S. Angeli, M. Panagi, T. Stylianopoulos, and F. Mpekris. 2024. 'Sonopermeation combined with stroma normalization enables complete cure using nano-immunotherapy in murine breast tumors', *Under Review*.

Netti, P. A., D. A. Berk, M. A. Swartz, A. J. Grodzinsky, and R. K. Jain. 2000. 'Role of extracellular matrix assembly in interstitial transport in solid tumors', *Cancer Res*, 60: 2497-503.

Panagi, M., F. Mpekris, C. Voutouri, A. G. Hadjigeorgiou, C. Symeonidou, E. Porfyriou, C. Michael, A. Stylianou, J. D. Martin, H. Cabral, A. Constantinidou, and T. Stylianopoulos. 2024. 'Stabilizing Tumor-Resident Mast Cells Restores T-Cell Infiltration and Sensitizes Sarcomas to PD-L1 Inhibition', *Clin Cancer Res*, 30: 2582-97.

Papageorgis, P., C. Polydorou, F. Mpekris, C. Voutouri, E. Agathokleous, C. P. Kapnissi-Christodoulou, and T. Stylianopoulos. 2017. 'Tranilast-induced stress alleviation in solid tumors improves the efficacy of chemo- and nanotherapeutics in a size-independent manner', *Sci Rep*, 7: 46140.

Perelson, A. S., D. E. Kirschner, and R. De Boer. 1993. 'Dynamics of HIV infection of CD4+ T cells', *Math Biosci*, 114: 81-125.

Pirentis, Athanassios P, Christiana Polydorou, Panagiotis Papageorgis, Chrysovalantis Voutouri, Fotios Mpekris, and Triantafyllos Stylianopoulos. 2015. 'Remodeling of extracellular matrix due to solid stress accumulation during tumor growth', *Connective tissue research*, 56: 345-54.

Plank, M. J., B. D. Sleeman, and P. F. Jones. 2004. 'The role of the angiopoietins in tumour angiogenesis', *Growth Factors*, 22: 1-11.

Pluen, A., Y. Boucher, S. Ramanujan, T. D. McKee, T. Gohongi, E. di Tomaso, E. B. Brown, Y. Izumi, R. B. Campbell, D. A. Berk, and R. K. Jain. 2001. 'Role of tumor-host interactions in interstitial diffusion of macromolecules: cranial vs. subcutaneous tumors', *Proc Natl Acad Sci U S A*, 98: 4628-33.

Popel, A. S. 1989. 'Theory of oxygen transport to tissue', *Crit Rev Biomed Eng*, 17: 257-321.

Rodriguez, E. K., A. Hoger, and A. D. McCulloch. 1994. 'Stress-dependent finite growth in soft elastic tissues', *J Biomech*, 27: 455-67.

Rolny, C., M. Mazzone, S. Tugues, D. Laoui, I. Johansson, C. Coulon, M. L. Squadrito, I. Segura, X. Li, E. Knevels, S. Costa, S. Vinckier, T. Dresselaer, P. Akerud, M. De Mol, H. Salomaki, M. Phillipson, S. Wyns, E. Larsson, I. Buysschaert, J. Botling, U. Himmelreich, J. A. Van Ginderachter, M. De Palma, M. Dewerchin, L. Claesson-Welsh, and P. Carmeliet. 2011. 'HRG

inhibits tumor growth and metastasis by inducing macrophage polarization and vessel normalization through downregulation of PlGF', *Cancer Cell*, 19: 31-44.

Roose, T., P. A. Netti, L. L. Munn, Y. Boucher, and R. K. Jain. 2003. 'Solid stress generated by spheroid growth estimated using a linear poroelasticity model', *Microvasc Res*, 66: 204-12.

Samani, A., J. Zubovits, and D. Plewes. 2007. 'Elastic moduli of normal and pathological human breast tissues: an inversion-technique-based investigation of 169 samples', *Phys Med Biol*, 52: 1565-76.

Schmidt, M. M., and K. D. Wittrup. 2009. 'A modeling analysis of the effects of molecular size and binding affinity on tumor targeting', *Molecular Cancer Therapeutics*, 8: 2861-71.

Schugart, R. C., A. Friedman, R. Zhao, and C. K. Sen. 2008. 'Wound angiogenesis as a function of tissue oxygen tension: a mathematical model', *Proc Natl Acad Sci U S A*, 105: 2628-33.

Skalak, R., S. Zargaryan, R. K. Jain, P. A. Netti, and A. Hoger. 1996. 'Compatibility and the genesis of residual stress by volumetric growth', *J Math Biol*, 34: 889-914.

Snipstad, S., E. Sulheim, C. de Lange Davies, C. Moonen, G. Storm, F. Kiessling, R. Schmid, and T. Lammers. 2018. 'Sonopermeation to improve drug delivery to tumors: from fundamental understanding to clinical translation', *Expert Opin Drug Deliv*, 15: 1249-61.

Stockmann, C., A. Doedens, A. Weidemann, N. Zhang, N. Takeda, J. I. Greenberg, D. A. Cheresh, and R. S. Johnson. 2008. 'Deletion of vascular endothelial growth factor in myeloid cells accelerates tumorigenesis', *Nature*, 456: 814-18.

Stylianopoulos, T., and R. K. Jain. 2013. 'Combining two strategies to improve perfusion and drug delivery in solid tumors', *Proc Natl Acad Sci U S A*, 110: 18632-37.

Stylianopoulos, T., J. D. Martin, M. Snuderl, F. Mpekris, S. R. Jain, and R. K. Jain. 2013. 'Coevolution of solid stress and interstitial fluid pressure in tumors during progression: Implications for vascular collapse', *Cancer research*, 73: 3833-41.

Stylianopoulos, T., A. Yeckel, J. J. Derby, X. J. Luo, M. S. Shephard, E. A. Sander, and V. H. Barocas. 2008. 'Permeability calculations in three-dimensional isotropic and oriented fiber networks', *Phys Fluids (1994)*, 20: 123601.

Taber, L. A. 2008. 'Theoretical study of Beloussov's hyper-restoration hypothesis for mechanical regulation of morphogenesis', *Biomech Model Mechanobiol*, 7: 427-41.

Tian, L., A. Goldstein, H. Wang, H. Ching Lo, I. Sun Kim, T. Welte, K. Sheng, L. E. Dobrolecki, X. Zhang, N. Putluri, T. L. Phung, S. A. Mani, F. Stossi, A. Sreekumar, M. A. Mancini, W. K. Decker, C. Zong, M. T. Lewis, and X. H. Zhang. 2017. 'Mutual regulation of tumour vessel normalization and immunostimulatory reprogramming', *Nature*, 544: 250-54.

Todaro, M., M. D'Asaro, N. Caccamo, F. Iovino, M. G. Francipane, S. Meraviglia, V. Orlando, C. La Mendola, G. Gulotta, A. Salerno, F. Dieli, and G. Stassi. 2009. 'Efficient killing of human colon cancer stem cells by gammadelta T lymphocytes', *J Immunol*, 182: 7287-96.

Tracqui, P. 1995. 'From passive diffusion to active cellular migration in mathematical models of tumour invasion', *Acta Biotheor*, 43: 443-64.

Tykodi, Scott S, Julie R Brahmer, Wen-Jen Hwu, Laura Q Chow, Suzanne Louise Topalian, Patrick Hwu, Kunle Odunsi, Luis H Camacho, John S Kauh, and Henry Clement Pitot. 2012. "PD-1/PD-L1 pathway as a target for cancer immunotherapy: Safety and clinical activity of BMS-936559, an anti-PD-L1 antibody, in patients with solid tumors." In.: American Society of Clinical Oncology.

Voutouri, C., N. D. Kirkpatrick, E. Chung, F. Mpekris, J. W. Baish, L. L. Munn, D. Fukumura, T. Stylianopoulos, and R. K. Jain. 2019. 'Experimental and computational analyses reveal dynamics of tumor vessel cooption and optimal treatment strategies', *Proc Natl Acad Sci U S A*, 116: 2662-71.

Voutouri, C., F. Mpekris, P. Papageorgis, A. D. Odysseos, and T. Stylianopoulos. 2014. 'Role of constitutive behavior and tumor-host mechanical interactions in the state of stress and growth of solid tumors', *PLoS One*, 9: e104717.

Voutouri, C., and T. Stylianopoulos. 2014. 'Evolution of osmotic pressure in solid tumors', *J Biomech*, 47: 3441-7.

Wang, Q., C. Liu, F. Zhu, F. Liu, P. Zhang, C. Guo, X. Wang, H. Li, C. Ma, W. Sun, Y. Zhang, W. Chen, and L. Zhang. 2010. 'Reoxygenation of hypoxia-differentiated dentritic cells induces Th1 and Th17 cell differentiation', *Mol Immunol*, 47: 922-31.

Wu, Darren C, and Clyde M Ofner. 2013. 'Adsorption and degradation of doxorubicin from aqueous solution in polypropylene containers', *AAPS PharmSciTech*, 14: 74-77.

Xu, G., P. V. Bayly, and L. A. Taber. 2009. 'Residual stress in the adult mouse brain', *Biomech Model Mechanobiol*, 8: 253-62.

Xu, G., P. S. Kemp, J. A. Hwu, A. M. Beagley, P. V. Bayly, and L. A. Taber. 2010. 'Opening angles and material properties of the early embryonic chick brain', *J Biomech Eng*, 132: 011005.
